# Characteristics of a novel treatment system for linear accelerator–based stereotactic radiosurgery

**DOI:** 10.1120/jacmp.v16i4.5313

**Published:** 2015-07-08

**Authors:** Ning Wen, Haisen Li, Kwang Song, Karen Chin‐Snyder, Yujiao Qin, Jinkoo Kim, Maria Bellon, Misbah Gulam, Stephen Gardner, Anthony Doemer, Suneetha Devpura, James Gordon, Indrin Chetty, Farzan Siddiqui, Munther Ajlouni, Robert Pompa, Zane Hammoud, Michael Simoff, Steven Kalkanis, Benjamin Movsas, M. Salim Siddiqui

**Affiliations:** ^1^ Department of Radiation Oncology Henry Ford Health System Detroit MI USA; ^2^ Department of Interventional Gastroenterology Henry Ford Health System Detroit MI USA; ^3^ Department of Thoracic Surgery Henry Ford Health System Detroit MI USA; ^4^ Department of Interventional Pulmonology Henry Ford Health System Detroit MI USA; ^5^ Department of Neurosurgery Henry Ford Health System Detroit MI USA

**Keywords:** commissioning, edge, six DoF couch, conical cones, end‐to‐end testing

## Abstract

The purpose of this study is to characterize the dosimetric properties and accuracy of a novel treatment platform (Edge radiosurgery system) for localizing and treating patients with frameless, image‐guided stereotactic radiosurgery (SRS) and stereotactic body radiotherapy (SBRT). Initial measurements of various components of the system, such as a comprehensive assessment of the dosimetric properties of the flattening filter‐free (FFF) beams for both high definition (HD120) MLC and conical cone‐based treatment, positioning accuracy and beam attenuation of a six degree of freedom (6DoF) couch, treatment head leakage test, and integrated end‐to‐end accuracy tests, have been performed. The end‐to‐end test of the system was performed by CT imaging a phantom and registering hidden targets on the treatment couch to determine the localization accuracy of the optical surface monitoring system (OSMS), cone‐beam CT (CBCT), and MV imaging systems, as well as the radiation isocenter targeting accuracy. The deviations between the percent depth‐dose curves acquired on the new linac‐based system (Edge), and the previously published machine with FFF beams (TrueBeam) beyond $D_{\text{max}}$ were within 1.0% for both energies. The maximum deviation of output factors between the Edge and TrueBeam was 1.6%. The optimized dosimetric leaf gap values, which were fitted using Eclipse dose calculations and measurements based on representative spine radiosurgery plans, were 0.700 mm and 1.000 mm, respectively. For the conical cones, 6X FFF has sharper penumbra ranging from 1.2−1.8 mm (80%‐20%) and 1.9−3.8 mm (90%‐10%) relative to 10X FFF, which has 1.2−2.2 mm and 2.3−5.1 mm, respectively. The relative attenuation measurements of the couch for PA, PA (rails‐in), oblique, oblique (rails‐out), oblique (rails‐in) were: −2.0%, −2.5%, −15.6%, −2.5%, −5.0% for 6X FFF and −1.4%, −1.5%, −12.2%, −2.5%, −5.0% for 10X FFF, respectively, with a slight decrease in attenuation versus field size. The systematic deviation between the OSMS and CBCT was −0.4±0.2 mm, 0.1±0.3 mm, and 0.0±0.1 mm in the vertical, longitudinal, and lateral directions. The mean values and standard deviations of the average deviation and maximum deviation of the daily Winston‐Lutz tests over three months are 0.20±0.03 mm and 0.66±0.18 mm, respectively. Initial testing of this novel system demonstrates the technology to be highly accurate and suitable for frameless, linac‐based SRS and SBRT treatment.

PACS number: 87.56.J‐

## I. INTRODUCTION

Since the term “stereotactic radiosurgery” was coined by Lars Leksell in 1951, there have been many technological, biological, and clinical advances in the field of stereotactic radiosurgery.^1^, ^2^, ^3^, ^4^ The accuracy of linear accelerators (linacs) has been improved significantly since the 1980s^5^, ^6^, ^7^ and linac‐based radiosurgery has been widely adopted over the subsequent decades. Since the 1990s, various technological advances have taken place to allow very precise treatments. The dedicated linacs have been designed exclusively for radiosurgery to further improve the targeting accuracy and high‐dose‐rate delivery. The mechanical isocenter accuracy of the C‐arm linac has reached submillimeter levels.^8^, ^9^ The flattening filter was first redesigned to be more efficient and later completely removed in order to deliver higher dose rates.^10^, ^11^ The multileaf collimators' (MLC) leaf resolution is also improving, with 2.5 mm leaf widths at the isocenter, in order to improve the dose conformality to the target.^(12)^ Treatment delivery methods have advanced to further improve conformality to complex geometric targets, while limiting dose to critical organs, such as dynamic conformal arc (DCA), Intensity Modulated Radiation Therapy (IMRT), and Volumetric Modulated Arc Therapy (VMAT).^13^, ^14^, ^15^, ^16^ In the era of image guidance, numerous methods have been developed for stereotactic treatment delivery, including optical surface monitoring, in‐room CT, stereoscopic X‐ray imaging, ultrasound, and cone‐beam computed tomography (CBCT).^17^, ^18^, ^19^, ^20^ Image‐guided frameless treatment has been systematically studied and the positioning accuracy has been validated for use in stereotactic treatments.^20^, ^21^

The latest platform for linac‐based SRS treatments (the Edge, Varian Medical Systems, Palo Alto, CA) offers multiple imaging modalities for treatment localization, including an optical surface monitoring system (OSMS) for surface tracking, 2.5 MV portal images for verification, automatically triggered monoscopic kV imaging to track intrafractional motion, 4D CBCT to evaluate tumor motion offline, extended CBCT images by stitching multiple CBCT scans together, and a Calypso/Varian electromagnetic beacon‐based tracking system. The new couch (PerfectPitch) supports six degrees of freedom (6DoF) corrections from multiple imaging modalities for precise patient setup. The flat panel imager is designed with a greater dynamic range, faster image readout rate, and a larger active area. This technology also has a stereotactic accessory package which includes conical cones ranging in diameter from 4 to 17.5 mm. Here we describe a comprehensive commissioning process suitable for modern, linac‐based SRS/SBRT with focus on the characterization of beam parameters, conical cones, 6DoF couch, dosimetric verification, and integrated end‐to‐end tests of this new technology.

## II. MATERIALS AND METHODS

### A. Flattening filter‐free (FFF) beam commissioning

Beam data were measured for the purpose of generating a beam model for the convolution/superposition dose algorithm (anisotropic analytical algorithm, AAA v 11.0.31 within the Eclipse Treatment Planning System (TPS), Varian Medical Systems). Measurements were performed for the two beam energies configured for our linac (flattening filter‐free photons, 6X FFF and 10X FFF). AAPM task group report No. 45 “AAPM Code of Practice for Radiotherapy Accelerators” recommendations were followed for commissioning tasks.^(22)^ Selection of different detectors for water phantom measurements were based on AAPM task group report No. 106 and small field dosimetry specification^(23)^ (Table 1). Field sizes ranged from $1\times 1\;{\text{cm}}^{2}$ to $40\times 40\;{\text{cm}}^{2}$ which were determined by the jaw (i.e., data were acquired with the MLCs parked). All mandatory and recommended beam data measurements (PDDs, cross‐plane and in‐plane profiles) were performed as specified in the Eclipse manual for commissioning the AAA algorithm beam model.

**Table 1:** Ion chambers and diodes used in the commissioning

*Ion Chamber*	*Active Volume* (${\text{cm}}^{3}$)	*Radius (mm)*	*Length (mm)*	*Central Electrode*	*Sensitivity (nC/Gy)*	*Tasks*
Scanditronix CC04	0.04	2.0	3.6 mm	C552	1.1	PDD, Profiles, OF $\geq 2\times 2\;{\text{cm}}^{2}$
Scanditronix CC01	0.01	1.0	3.6 mm	Steel	0.3	OF – Conical Cones
PTW PinPoint (31014)	0.015	1.0	5.0 mm	Aluminum	0.4	OF – Conical Cones
*Diode*	*Thickness of Active Volume (mm)*	*Diameter*	*Geometry – Active Area*	*Misc*.	*Sensitivity (nC/Gy)*	*Tasks*
Scanditronix SFD	0.06	0.6 mm	Circle	p‐type Unshielded	5.9	PDD, Profiles, OF $\leq 3\times 3\;{\text{cm}}^{2}$ and Conical Cones
Scanditronix PFD	0.06	2.0 mm	Circle	p‐type Shielded	33.3	OF – Conical Cones
Sun Nuclear EDGE	0.0025	$0.8\times 0.8\;{\text{mm}}^{2}$	Square	n‐type Unshielded	32.0	OF – Conical Cones

#### A.1 Percent depth dose and profiles

PDDs and profiles were scanned for ten different field sizes, ranging from 1×1 to $40\times 40\;{\text{cm}}^{2}$ at an SSD of 100 cm. The central electrode of the chamber was oriented parallel to the in‐plane direction, perpendicular to the beam axis. The effective point of measurement correction was applied during the beam scanning since the AAA does not perform this correction automatically. Cross‐plane and in‐plane profiles were acquired at five different depths ($d_{\text{max}}$, 5, 10, 20, and 30 cm) for each field size. PDD and profiles curves were measured with a CC04 cylindrical chamber (Scanditronix Wellhofer, IBA Dosimetry America, Barlett, TN) for field sizes equal or greater than $2\times 2\;{\text{cm}}^{2}$ using the 400 MU/min dose rate. The SFD (Scanditronix Wellhofer) was used for field sizes $1\times 1\;{\text{cm}}^{2}$ and $2\times 2\;{\text{cm}}^{2}$. These curves were used for our own small field dosimetry evaluation since the profile or PDD curves for field sizes smaller than $2\times 2\;{\text{cm}}^{2}$ are not used by the beam configuration in Eclipse.^(24)^ A reference detector was not used for the diode measurement. Data were acquired with the field detector in a step‐by‐step mode, with data sampled at every 0.3 mm. The beams were scanned at the maximum dose rate and the acquisition sampling was set to improve the signal‐to‐noise ratio.^(23)^ Both PDD and profile curves were compared to data acquired from other linacs in our clinic with FFF beam configurations (TrueBeam linacs, Varian Medical Systems).^(9)^

The linearity response with dose rate of the CC04 chamber was measured for 6X FFF (range: 400–1400 MU/min) and 10X FFF (range: 400–2400 MU/min) with a fixed MU. The ion chamber collection efficiency was also measured for both energies at the maximum dose rate for field sizes of 10×10 and $15\times 15\;{\text{cm}}^{2}$. The two‐voltage method (300 V and 150 V) was used to calculate the recombination correction factor ($P_{\text{ion}}$) at the central axis and one off‐axis position (2.4 and 5.6 cm off‐axis, transverse plane) for each field size.

#### A.2 Output factors (OFs)

Total scatter factors ($S_{\text{cp}}$) were acquired at 95 cm SSD and 5 cm depth using a CC04 ion chamber at field sizes ranging from 3×3 to $40\times 40\;{\text{cm}}^{2}$. The SFD was used for field sizes from 1×1 to $3\times 3\;{\text{cm}}^{2}$. The diode was cross calibrated with the CC04 at $3\times 3\;{\text{cm}}^{2}$ as follows:

(1) $$\frac{SFD(fs)}{SFD(3\times 3)}\times \frac{CC04(3\times 3)}{CC04(10\times 10)}$$

where *SFD(fs)* is the diode reading for the small field size, *SFD*(3×3) is the diode reading for the $3\times 3\;{\text{cm}}^{2}$ field, *CC04*(3×3) is the reading of the CC04 chamber for the $3\times 3\;{\text{cm}}^{2}$ field, and *CC04*(10×10) is the reading of the CC04 chamber for the field size $10\times 10\;{\text{cm}}^{2}$.

#### A.3 MLC Leaf transmission and dosimetric leaf gap (DLG) measurements

The MLC leaf transmission and DLG were commissioned as follows. The baseline values were measured through extrapolation to a leaf gap of zero on a plot of dose as a function of the gap between opposite leaves.^(25)^ The values were then iteratively adjusted using three representative spine radiosurgery plans (vertebral body, paraspinal mass, and spinous process) for the purpose of optimizing agreement between calculations and measurements for both IMRT and RapidArc techniques. Point doses were measured using a PTW PinPoint chamber 31014 (PTW, Freiburg GmbH, Germany) in a Lucy phantom (Standard Imaging Inc., Middleton, WI). Planar doses were measured using Gafchromic EBT3 films (International Specialty Products, Wayne, NJ) sandwiched at the center of a 10 cm thick acrylic phantom (BrainLAB, Feldkirchen, Germany).

### B. Conical cones commissioning

The Edge conical collimator accessory system consists of seven circular cones, 4, 5, 7.5, 10, 12.5, 15, 17.5 mm in diameter. The cones are inserted in an accessory mount that attaches to the collimator face plate, with an Integrated Conical Collimator Verification & Interlock (ICVI) system which recognizes a specific cone during mounting and dismounting. PDD data were acquired at SSD of 100 cm using the SFD and converted to TMR values using the standard conversion method.^(26)^ The off‐axis profiles were scanned in both in‐plane and cross‐plane directions at the depth of 5 cm at three SSDs: 80, 90, and 100 cm. Output factors (OFs) for all cones were measured with a $5\times 5\;{\text{cm}}^{2}$ jaw size at 95 cm SSD and 5 cm depth for both 6X FFF and 10X FFF modes using five different detectors (Table 1): Edge diode (Sun Nuclear, Melbourne, FL), SFD, PFD (Scanditronix Wellhofer), CC01 chamber, and PinPoint chamber 31014. All the diodes were cross‐calibrated with the CC04 at the $3\times 3\;{\text{cm}}^{2}$ field size. Results were compared with the manufacturer representative data measured with the Edge diode.

### C. Six degree of freedom (6DoF) couch commissioning

Couch commissioning procedures included positioning accuracy of the imaging system and couch to detect linear and rotational offsets, rigidity test of the couch insert in the lateral direction with both rails at the center (‘in’ position), and attenuation measurements of the rails and inserts.

#### C.1 6DoF positioning accuracy

The accuracy of the couch position readout of each of the six axes was validated at various positions with and without a RANDO pelvic phantom (13.8 kg) (The Phantom Laboratory, Salem, NY) placed on the couch. The positional readout (PRO) accuracy was verified at ten positions (±1,±2,±5,±10,±20 cm) using a tape measure in each translational direction, four positions (45°,90°,315°,270°) using a protractor in the yaw direction, and seven positions (0°,± 1°, ± 2°, ± 3°) using a digital level in the pitch and roll direction. The pitch and roll positioning uncertainties of the online image registration were evaluated using the OSMS QA phantom (Vision RT, London, UK) with and without the RANDO phantom to evaluate the weight factor. The central BB in the phantom was aligned to the isocenter using MV/KV orthogonal pair imaging. A given pitch and roll were applied (+3°/+3°,−3°/−3°, and 0°/0°), a MV/KV image pair was taken, and the distance between the center of the BB and isocenter was measured to evaluate the pitch and roll positioning accuracy.

#### C.2 Rigidity test of couch insert

The rigidity test was performed at two couch positions in the longitudinal direction with a volunteer (96.2 kg) lying on the couch. The volunteer was positioned at the center of the Calypso‐compatible couchtop insert and the couch was also centered laterally. A 3° pitch and roll was applied to the couch. The pitch angle was given to evaluate the potential influence on the roll. A digital level was used to check for possible angular deviation at the longitudinal end of the couch insert. The couch rigidity in roll angle with respect to the couch position in the lateral direction was also tested by off‐centering the volunteer to the maximum lateral direction at 24.8 cm.^(27)^

#### C.3 Beam attenuation through the couch top and rails

The couch top consists of two mobile, Kevlar support rails, a nonconductive Kevlar Varian/Calypso insert, and a solid carbon fiber KVue insert. Prior to installation of the linac, both Calypso and KVue inserts along with the support rails were CT scanned with the rails at various positions. An additional scan with the couch top 15 cm above the CT table top was obtained with 20 cm solid water to mimic patient‐like setups. The attenuation measurements were obtained for field sizes of 2, 4, and $10\;{\text{cm}}^{2}$ at 42 gantry angles, including six pairs of opposing fields and other oblique angles in which the beams traversed the couch inserts and/or rails. The results were then used to determine an accurate structure model for the planning system.

### D. IMRT and RapidArc commissioning

**Figure 1:**
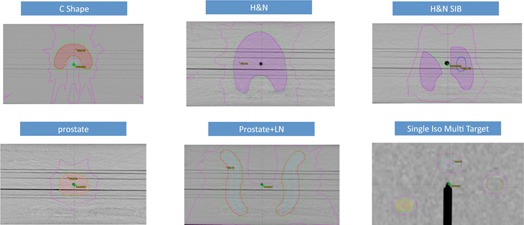
C‐shape target planned with IMRT using 6X FFF; head and neck PTV target with the cord and parotid glands planned with IMRT using 6X FFF; head and neck SIB plan: HN PTV50 (shaded magenta) and PTV60 (blue) targets with the cord and parotid glands planned with IMRT using 6X FFF; prostate PTV (pink) planned with rectum and bladder with IMRT using 6X FFF; prostate+LN plan: prostate+LN (blue) PTV target (red) with rectum and bladder planned with IMRT using 6X FFF; single Iso multitarget plan: three targets (orange, purple, and red) planned with IMRT using 6X FFF. The isodose lines represent 95% (green) and 50% (magenta) prescription dose.

A total of 21 plans generated using updated AAPM TG 119 test suite^(28)^ were planned and calculated with the AAA, V.11.0.31 algorithm in the Eclipse TPS. A Solid Water phantom (density: $1.03\;g/{\text{cm}}^{3}$) was used to evaluate the dosimetric accuracy of both energies using the maximum dose rate. The actual dose rate varied during the delivery for the RapidArc plans. The 6DoF couch top, with the rails in the ‘out’ position, was included in the dose calculation. The 21 treatment plans included hard C shape, head and neck, head and neck with simultaneous integrated boost, prostate, prostate and lymph nodes, and single isocenter multiple intracranical targets (SIMT) (Fig. 1). All IMRT cases used seven to nine beams and RapidArc cases used two arcs, except for the SIMT case, which used four arcs, with dose optimization constraints that follow the technique of Clark et al..^(29)^ Point dose measurement using an ion chamber (PTW PinPoint Chamber, Model 31014) and planar dose distribution measurement using films (Gafchromic EBT3) were performed in both the high‐dose target and a low‐dose region. For the SIMT case, the distance between the isocenter and the center of each of three targets was 2, 4, and 4.5 cm respectively and 16 Gy was delivered to each target. Ion chamber measurements were made at the isocenter and the center of one of the targets 2 cm away. Film was delivered in the axial plane 1 cm posterior to the isocenter. An in‐house software was developed to integrate Gafchromic film dosimetry protocol using EBT3 films which streamlines a dose pattern delivery for calibration, calibration curve fitting, film scanning in the fixed scanner position, dose mapping from multiple color channels, rigid registration between the calculated and measured dose plans based on the intensity levels, and profile/gamma analysis.^(30)^

Twelve SRS/SBRT patient plans were measured using the same setup. The treatment sites included intracranial, spine, lung, pancreas, liver, and adrenal gland lesions. Table 2 shows the detail of the plans, including treatment fractions, prescription dose, tumor volume size, conformity index,^(31)^ gradient index,^(32)^ and maximum plan dose. The isocenter dose was measured with ion chamber, and the coronal planar dose distribution was measured with EBT3 films.

**Table 2:** The treatment plan and QA results of 12 SRS/SBRT patient cases

*Case No*	*Treatment Site*	*Fractions*	*Prescription Dose (Gy)*	*Treatment Technique*	*Energy*	*Volume Size (cc)*	*Conformity Index*	*Gradient Index*	*Max Dose (Gy)*	*Point Dose Difference*	*Gamma* 3%/3 mm	*Gamma* 3%/1 mm
1	Intracranial, Left Temporal	1	16	RapidArc	6X FFF	20.1	0.96	3.78	19.75	−0.1%	100.0%	98.9%
2	Intracranial, Right Mid Cranial Fossa	3	10	RapidArc	6X FFF	61.8	1.10	3.70	35.16	−2.1%	99.9%	90.8%
3	Intracranial, GBM	4	32	RapidArc	6X FFF	71.0	0.93	4.27	39.95	1.7%	100.0%	99.4%
4	Spine, L2	1	18	RapidArc	10X FFF	54.0	1.02	4.90	22.32	−0.5%	99.2%	91.0%
5	Spine, T5 Epidural	1	18	IMRT	6X FFF	14.4	1.01	4.09	20.39	−0.9%	99.3%	86.6%
6	Spine, C2‐C3	1	16	RapidArc	6X FFF	26.1	1.02	3.83	17.78	0.8%	100.0%	99.5%
7	Pancreas	5	35	IMRT	10X FFF	46.2	1.02	5.21	36.23	0.1%	100.0%	98.1%
8	Liver	5	35	RapidArc	10X FFF	239.8	1.00	3.58	39.11	2.1%	99.6%	87.0%
9	Left Adrenal	5	35	RapidArc	6X FFF	12.8	1.00	4.38	37.63	1.2%	97.3%	80.9%
10	Lung, Right Upper Lobe	4	48	RapidArc	6X FFF	17.8	0.98	3.45	52.33	2.6%	99.4%	97.9%
11	Lung, Left Lower Lobe	4	48	IMRT	6X FFF	60.7	1.01	3.89	56.47	−1.8%	98.5%	94.0%
12	Lung, Right Upper Lobe	4	48	RapidArc	6X FFF	22.8	0.99	4.62	51.89	−2.2%	99.4%	92.2%

### E. The end‐to‐end test

**Figure 2:**
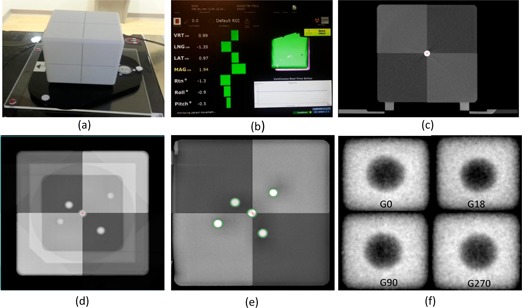
The OSMS QA phantom (a) sitting on top of an acrylic base plate. The localization (b) of phantom surface using the OSMS system. The difference (delta) between the current position of the OSMS phantom and its reference position is shown in 6DoF. The six degree automatic fusion (c) between planning CT and CBCT after adjusting the contrast of the acquired image and reference image to achieve optimal visualization of the BBs. An orthogonal MV (d)/KV (e) image set is taken and 2D–3D image fusion is performed to quantify the residual error. Four representative (f) MLC defined portal images of the Winston‐Lutz test.

Daily end‐to‐end quality assurance tests were performed to assess the overall accuracy of the system from CT simulation, treatment planning, image‐based localization, and final treatment delivery using the OSMS QA phantom. The phantom is a polystyrene $15\times 15\times 15\;{\text{cm}}^{3}$ cube embedded with five 7.5 mm diameter ceramic BBs (Fig. 2(a)). One of the BBs was located at the center of the cube. The phantom was scanned with 0.8 mm slice thickness (pixel size $0.6\times 0.6\;{\text{mm}}^{2}$) without the base plate. The cube and BBs were contoured in Eclipse and used as the reference image. In the treatment room, the phantom was set up on top of an acrylic base plate and fixed to the pegs of an indexing bar for consistent setup. The acrylic plate was engraved with three notches in which the three screws of the OSMS phantom holder were seated. The couch was set at a fixed position (vertical: 10.0 cm; longitudinal: 98.5 cm; lateral: 0.0 cm, pitch: 0.5°, and roll: 0.5°). The OSMS system was first used to localize the phantom surface, and the difference (delta) between the current position of the OSMS phantom and its reference position was recorded (Fig. 2(b)). CBCT images of the phantom (kV=100; mAs=265, 1 mm slice thickness, full fan) were acquired, and automatic fusion was performed after adjusting the contrast of the acquired image and reference image to achieve optimal window and leveling in order to visualize the BBs (Fig. 2(c)). Six dimensional (6D) fusion shifts were recorded and applied. The phantom position in the OSMS system after correction was recorded to evaluate the residual error. An orthogonal MV/KV set was taken and 2D–3D image fusion was performed to quantify the residual error (Figs. 2(d) and (e))). An electronic portal imaging device (EPID)‐based Winston‐Lutz (WL) test was then performed to verify the isocenter targeting accuracy. Twelve $2\times 2\;{\text{cm}}^{2}$, MLC‐defined portal images were acquired at four gantry, four couch, and four collimator angles, which were analyzed by an in‐house developed C++ software based on an open‐source framework (Insight Segmentation and Registration Toolkit 4.3.2) to measure the distance between the center of the central BB and the full width at half maximum (FWHM) of the radiation field (Fig. 2(f)). The coincidence of the imaging systems and radiation isocenter are evaluated on a daily basis, according to AAPM TG 142 recommendation.^(33)^

Independent end‐to‐end tests were performed using the Imaging and Radiation Oncology Core (IROC‐Houston) spine and thorax phantoms. The phantoms were scanned, treatment planned, and irradiated at our institution, according to the IROC‐Houston credentialing criteria. After irradiation, the phantoms were sent back to IROC‐Houston, where absolute point dose was measured with TLDs and 2D film dose planes were measured with Gafchromic EBT2 film, analysis was completed independently by IROC‐Houston. Treatment plans were generated with the Eclipse TPS using the same AAA algorithm and delivered using the RapidArc technique for the spine phantom and IMRT for the thorax phantom. Both phantoms were localized using the OBI system, where CBCT was used for initial setup.

The spine phantom consists of a pentagon shaped PTV (42 cc) abutting bone and a cylindrical spinal cord structure; the PTV is set between the right and left lung structures. The spine phantom has four TLDs within the PTV structure in the high‐dose region and one within the heart in the low‐dose region. Two films bisect the PTV in the axial and sagittal planes. The thorax phantom consists of an ellipsoidal‐shaped PTV (72 cc) located in the middle of a cylindrical volume of lung. The thorax phantom contains two TLDs within the PTV, and two TLDs in the low‐dose region, one in the heart and one in the cord. Three films bisect the PTV in the axial, coronal, and sagittal planes.

### F. Treatment head leakage test

**Figure 3:**
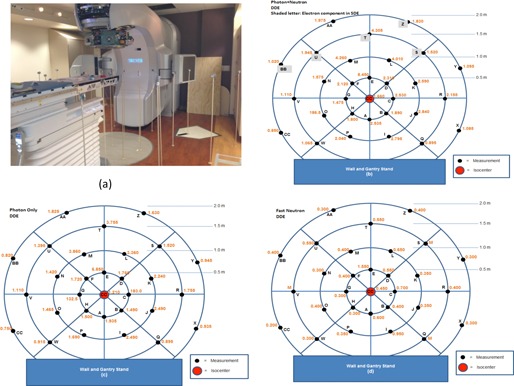
The placement (a) of the T series dosimeters around a 2 m radius circular plane. The deep dose equivalent map of photon and neutron combined (b), photon only (c), and fast neutron only (d). The maximum measured head leakage dose was 8.45, 6.85, and 1.55 mSv, respectively, all located at point E, 0.5 m toward the couch direction.

Treatment head leakage was measured using 30 pairs of Luxel+ T series dosimeters (Landauder, Glenwood, IL) placed around a 2 meter radius circular plane, in a plane perpendicular to the beam axis at the isocenter. Figure 3(a) shows the placement of each pair of dosimeters. Ten thousand (10,000) MUs were delivered to the dosimeters at gantry 0° position, with both MLC and jaw at most closed position, using the highest energy, 10X FFF, at 2400 MU/min. The average reading of each pair of dosimeters was recorded.

### G. Developer mode

The Edge system includes Developer Mode, enabling the use of XML scripting for automation of commissioning and QA procedures. XML scripting was used for various commissioning tasks including beam scanning, couch modeling, and end‐to‐end tests.

## III. RESULTS

### A. Beam commissioning

#### A.1 Percent depth dose and profile evaluation

**Figure 4:**
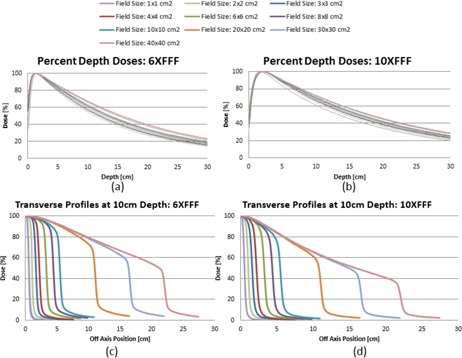
PDD curves normalized at $D_{\text{max}}$ for 6X FFF (a) and 10X FFF (b) for the field sizes ranging from 1×1 to $40\times 40\;{\text{cm}}^{2}$. The cross‐plane profiles measured at 10 cm depth for all 10 field sizes for 6X FFF (c) and 10X FFF (d). A CC04 cylindrical chamber was used for field sizes greater than $2\times 2\;{\text{cm}}^{2}$ using the 400 MU/min dose rate, and the SFD was used for field sizes $1\times 1\;{\text{cm}}^{2}$ and $2\times 2\;{\text{cm}}^{2}$ using the maximum dose rate. The curves are normalized to 100% on the central axis.

Figure 4 shows the PDD curves normalized at $D_{\text{max}}$ for 6X FFF (a) and 10X FFF (b) for the field sizes ranging from 1×1 to $40\times 40\;{\text{cm}}^{2}$. Table 3 summarizes the $D_{\text{max}}$ and PDD values at 5, 10, 20, and 30 cm depth. The deviations between the photon beam curves acquired on the new linac‐based system (Edge) and the previously published machine with FFF beams (TrueBeam) beyond $D_{\text{max}}$ were within 1.0% for both energies. The beam quality specifier ($\%\text{dd}{\left(10\right)}_{\times }$) for the Edge was 63.0% and 70.6% for 6X FFF and 10X FFF, respectively without 1 mm lead foil. With a 1 mm lead foil, $\%\text{dd}{\left(10\right)}_{\times }$ increased to 71.1% for 10X FFF; however, the difference between the quality conversion factors ($k_{Q}$) for 10X FFF were within 0.1% with and without the lead foil.

**Figure 5:**
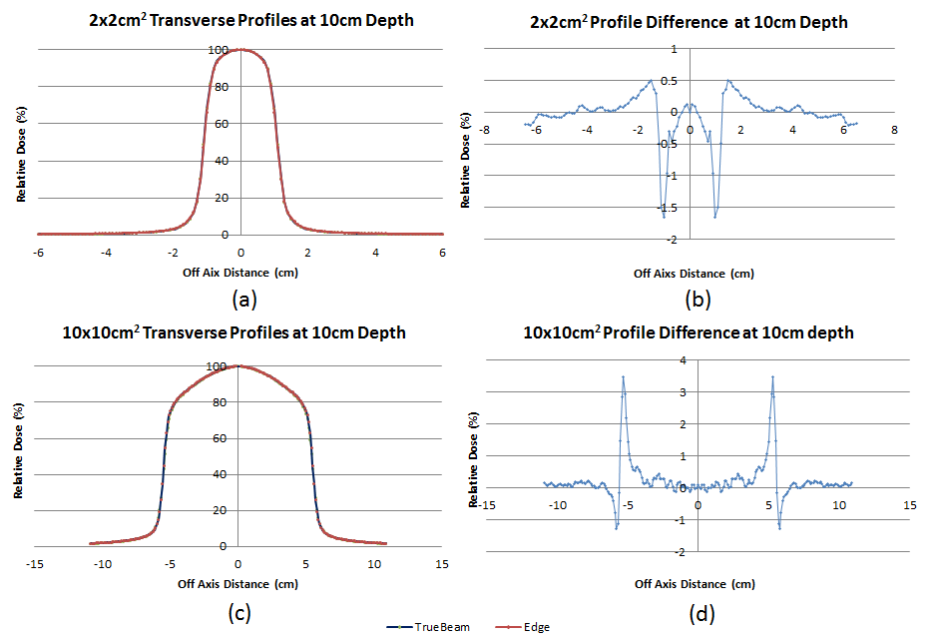
Comparison of profile curves between the Edge and the TrueBeam for two representative fields using 10X FFF: $2\times 2\;{\text{cm}}^{2}$ and $10\times 10\;{\text{cm}}^{2}$. The profiles between the Edge and the TrueBeam were practically the same, with slightly sharper penumbra obtained on the Edge at all the depths.

Figures 4(c) and (d) illustrate the cross‐plane profiles measured at 10 cm depth for all ten field sizes from 1×1 to $40\times 40\;{\text{cm}}^{2}$. The curves are normalized to 100% on the central axis. Since only FFF modes were commissioned for the Edge, we could not use the penumbra normalization method proposed by Pönisch et al.^(34)^ Figure 5 shows direct comparison of profile curves between the Edge and the TrueBeam for two representative fields using 10X FFF: $2\times 2\;{\text{cm}}^{2}$ and $10\times 10\;{\text{cm}}^{2}$. The profiles between the Edge and the TrueBeam were practically the same, with slightly sharper penumbra obtained on the Edge at all the depths.

The values of $P_{\text{ion}}$ at the central axis and two off‐axis positions were compared. The output constancy was within 0.1% with various dose rates for both energies. The ion chamber collection efficiency off‐axis agreed within 0.3% of the values at the central axis for the two field sizes evaluated.

**Table 3:** $D_{\text{max}}$ and PDD values at 5, 10, 20, and 30 cm depth for 6X FFF and 10X FFF

Energy Field Size		1×1	2×2	3×3	4×4	5×5	6×6	8×8	10×10	12×12	15×15	20×20	30×30	35×35	40×40
6X FFF	$D_{\text{max}}$ (cm)	1.10	1.30	1.34	1.40	1.35	1.24	1.30	1.35	1.36	1.35	1.18	1.27	1.21	1.20
	5 cm (%)	75.8	78.5	79.9	81.2	81.8	82.4	83.3	84.2	84.4	85.2	85.3	85.8	85.9	86.2
	10 cm (%)	51.7	54.1	56.2	57.8	58.9	59.9	61.5	63.0	63.8	65.0	65.8	66.8	67.2	67.3
	20 cm (%)	25.5	26.8	28.7	29.7	30.7	31.4	32.9	34.4	35.1	36.6	37.8	39.2	39.5	39.6
	30 cm (%)	13.2	14.1	15.3	15.9	16.4	17.0	17.9	18.9	19.7	20.5	21.6	22.9	23.0	23.2
10X FFF	$D_{\text{max}}$(cm)	1.80	2.07	2.20	2.20	2.06	2.14	2.14	2.36	2.06	1.97	2.19	1.93	1.95	2.11
	5 cm (%)	83.5	87.0	88.0	88.5	89.1	89.5	89.8	90.2	90.1	90.3	90.5	90.3	90.3	90.3
	10 cm (%)	61.2	64.3	66.3	67.2	68.2	68.7	69.5	70.6	71.0	71.4	72.0	72.2	72.5	72.5
	20 cm (%)	34.4	36.2	38.0	39.0	39.6	40.2	41.5	42.6	43.3	43.8	44.8	45.3	45.6	45.8
	30 cm (%)	20.0	21.4	22.8	23.3	23.8	24.2	25.2	25.9	26.6	26.8	27.9	28.6	28.8	29.1

#### A.2 Output factors

**Figure 6:**
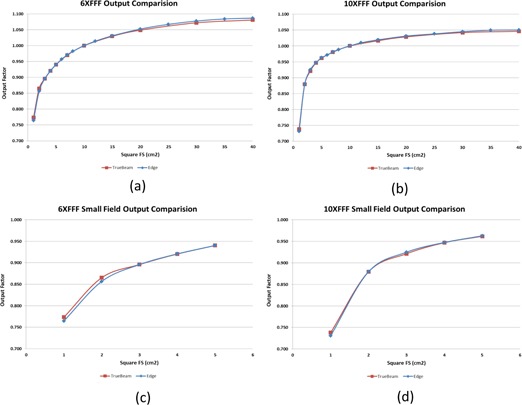
Comparison of output factors between the Edge and TrueBeam for symmetrical fields ranging from $1\times 1\;{\text{cm}}^{2}$ to $40\times 40\;{\text{cm}}^{2}$ for 6X FFF (a) and 10X FFF (b). The figures are magnified for small field sizes in (c) and (d).

The output factors $S_{\text{cp}}$ for the symmetrical fields and rectangular fields are tabulated in 4, 5 for 6X FFF and 10X FFF, respectively. The shielded area in the table corresponds to data measured with the SFD detector. $S_{\text{cp}}$ for symmetrical fields ranging from 1×1 to $40\times 40\;{\text{cm}}^{2}$ were also plotted in Fig. 6 and compared against the TrueBeam machine (Fig. 6(b)). The maximum deviation between the Edge and TrueBeam was 1.6% for field size of $1\times 2\;{\text{cm}}^{2}$ (6X FFF) and 1.0% for $1\times 1\;{\text{cm}}^{2}$ (10X FFF).

**Table 4:** Output factors measured with CC04 and SFD for 6X FFF

*Y\X*	*1* ^a^	*2* ^a^	*3*	*4*	*5*	*6*	7	*8*	*10*	*12*	*15*	*20*	*25*	*30*	*35*	*40*
1	0.765	0.799	0.808^a^	0.811^a^	0.815^a^	0.818^a^	0.818^a^	0.821^a^	0.821^a^	0.822^a^	0.825^a^	0.825^a^	0.826^a^	0.827^a^	0.827^a^	0.828^a^
2	0.806	0.856	0.872^a^	0.881^a^	0.887^a^	0.892^a^	0.896^a^	0.899^a^	0.901^a^	0.904^a^	0.907^a^	0.909^a^	0.910^a^	0.911^a^	0.912^a^	0.913^a^
3	0.817	0.874	0.896	0.907	0.913	0.919	0.922	0.925	0.928	0.930	0.933	0.935	0.937	0.938	0.937	0.937
4	0.823	0.885	0.907	0.921	0.929	0.935	0.940	0.943	0.947	0.950	0.953	0.957	0.959	0.960	0.960	0.959
5	0.826	0.891	0.916	0.930	0.940	0.947	0.953	0.958	0.962	0.965	0.969	0.974	0.977	0.977	0.977	0.977
6	0.828	0.897	0.922	0.938	0.949	0.957	0.963	0.968	0.973	0.978	0.982	0.988	0.990	0.991	0.992	0.991
7	0.831	0.901	0.926	0.944	0.955	0.964	0.971	0.976	0.982	0.987	0.992	0.998	1.002	1.003	1.004	1.003
8	0.832	0.904	0.929	0.949	0.960	0.969	0.977	0.982	0.989	0.995	1.000	1.007	1.010	1.012	1.013	1.012
10	0.835	0.909	0.934	0.955	0.967	0.978	0.986	0.991	1.000	1.006	1.012	1.020	1.025	1.027	1.028	1.027
12	0.836	0.912	0.938	0.958	0.972	0.983	0.992	0.998	1.008	1.014	1.022	1.029	1.035	1.038	1.039	1.038
15	0.839	0.915	0.940	0.962	0.975	0.988	0.998	1.004	1.015	1.023	1.031	1.041	1.047	1.050	1.051	1.051
20	0.840	0.919	0.943	0.966	0.981	0.992	1.003	1.010	1.023	1.030	1.040	1.052	1.059	1.063	1.065	1.064
25	0.841	0.920	0.945	0.968	0.983	0.996	1.006	1.015	1.027	1.035	1.047	1.059	1.067	1.072	1.074	1.073
30	0.843	0.922	0.947	0.970	0.986	0.999	1.009	1.018	1.031	1.041	1.052	1.065	1.073	1.078	1.080	1.080
35	0.844	0.923	0.947	0.971	0.987	1.000	1.012	1.020	1.034	1.043	1.055	1.070	1.078	1.082	1.084	1.085
40	0.843	0.924	0.948	0.972	0.988	1.001	1.013	1.022	1.036	1.045	1.057	1.072	1.079	1.084	1.086	1.087

a ^a^ The data measured by SFD.

**Table 5:** Output factors measured with CC04 and SFD for 10X FFF

*Y\X*	*1* ^a^	*2* ^a^	*3*	*4*	*5*	*6*	7	*8*	*10*	*12*	*15*	*20*	*25*	*30*	*35*	*40*
1	0.731	0.784	0.796^a^	0.801^a^	0.800^a^	0.803^a^	0.804^a^	0.804^a^	0.806^a^	0.805^a^	0.807^a^	0.807^a^	0.808^a^	0.809^a^	0.808^a^	0.809^a^
2	0.800	0.880	0.897^a^	0.906^a^	0.908^a^	0.912^a^	0.914^a^	0.914^a^	0.916^a^	0.919^a^	0.922^a^	0.921^a^	0.924^a^	0.923^a^	0.924^a^	0.924^a^
3	0.814	0.900	0.925	0.935	0.941	0.944	0.945	0.947	0.949	0.952	0.952	0.952	0.955	0.955	0.954	0.953
4	0.819	0.911	0.935	0.947	0.954	0.957	0.961	0.964	0.966	0.969	0.970	0.970	0.972	0.972	0.971	0.972
5	0.821	0.916	0.942	0.955	0.963	0.967	0.970	0.973	0.977	0.979	0.980	0.983	0.983	0.984	0.983	0.984
6	0.824	0.920	0.945	0.960	0.968	0.972	0.976	0.980	0.984	0.987	0.987	0.990	0.992	0.993	0.993	0.994
7	0.825	0.922	0.949	0.963	0.973	0.977	0.981	0.986	0.990	0.993	0.995	0.997	0.999	1.000	1.001	1.000
8	0.826	0.923	0.950	0.966	0.976	0.981	0.984	0.989	0.994	0.998	1.001	1.003	1.004	1.006	1.004	1.006
10	0.829	0.926	0.953	0.970	0.982	0.985	0.992	0.995	1.000	1.004	1.008	1.012	1.013	1.016	1.014	1.015
12	0.828	0.928	0.956	0.972	0.984	0.988	0.995	0.998	1.005	1.010	1.012	1.018	1.021	1.022	1.022	1.024
15	0.832	0.930	0.958	0.974	0.986	0.991	0.999	1.004	1.011	1.025	1.019	1.024	1.028	1.029	1.028	1.028
20	0.834	0.933	0.960	0.978	0.988	0.996	1.003	1.008	1.015	1.020	1.024	1.031	1.034	1.037	1.036	1.038
25	0.833	0.935	0.961	0.980	0.992	0.998	1.004	1.009	1.018	1.023	1.029	1.034	1.038	1.043	1.041	1.042
30	0.832	0.935	0.964	0.980	0.994	0.999	1.006	1.013	1.020	1.028	1.032	1.039	1.043	1.045	1.045	1.047
35	0.833	0.936	0.964	0.981	0.995	1.002	1.009	1.014	1.022	1.028	1.033	1.042	1.045	1.048	1.050	1.050
40	0.837	0.938	0.965	0.983	0.995	1.003	1.010	1.015	1.023	1.029	1.034	1.042	1.048	1.051	1.051	1.050

a ^a^ The data measured by SFD.

#### A.3 HDMLC transmission and DLG

The measured DLG values were 0.507 mm for 6X FFF and 0.622 mm for 10X FFF. Optimized values, which were fitted using Eclipse dose calculations and measurements based on representative spine radiosurgery plans, were 0.700 mm and 1.000 mm, respectively. The MLC transmission values were 1.209% for 6X FFF and 1.427% for 10X FFF. Dose difference ratios of ion chamber measurements were 0.015%±0.008% for 6X FFF and 0.010%±0.010% for 10X FFF, and the passing rates for 2%/2 mm gamma criteria were 98.0±1.0 for 6X FFF and 96.9±1.9 for 10X FFF after the DLG optimization.

### B. Conical cones

**Figure 7:**
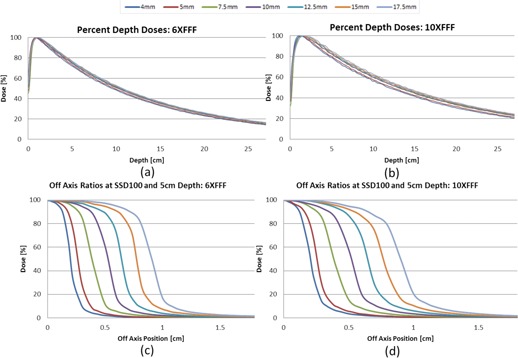
PDD curves normalized at $D_{\text{max}}$ for 6X FFF (a) and 10X FFF (b) for the conical cones ranging from 4 mm to 17.5 mm. The off‐axis ratio measured at 5 cm depth, 100 cm SSD for 6X FFF (c) and 10X FFF (d). The curves are normalized to 100% on the central axis.

**Figure 8:**
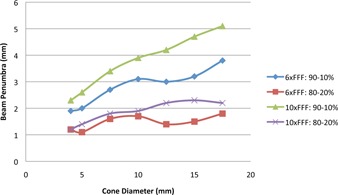
The beam penumbra (width between 90%‐10% and 80%‐20%) increases as the diameter of the cone increases for both energies. The beam penumbra increases faster for the 90%‐10% value than for the 80%‐20% value.

Figure 7(a) and (b) shows the PDD data for the conical cones for 6X FFF and 10X FFF. The off‐axis ratios for all the conical cones at the depth of 5 cm at 100 cm SSD are shown in Fig. 7(c) and (d). All beam profile data were normalized to the central axis. The beam penumbra (width between 90%‐10% and 80%‐20%) increases as the diameter of the cone increases, as shown in Fig. 8. 6X FFF has sharper penumbra ranging from 1.2−1.8 mm (80%‐20%) and 1.9−3.8 mm (90%‐10%) relative to 10X FFF, which has 1.2−2.2 mm and 2.3−5.1 mm, respectively.

Table 6 shows the OFs of the cones using the Edge detector, with and without cross‐calibration, at an intermediate field size. Because the Edge detector is independent of variation in energy spectrum,^(35)^ minimal difference between the two measurements was observed (OFs were within 0.2% and 0.7% for 6X FFF and 10X FFF, respectively).

The percent difference between OFs we measured using different detectors and the data from the manufacturer measured with the Edge detector (available at the Vendor website) is also shown in Table 6. The difference was ~1% for the Edge detector and increased to 4% for the SFD detector. As observed in Table 6, the PFD, CC01, and PinPoint ion chambers show much lower OFs for the smaller cones due to the volume averaging effect.

**Table 6:** Output factors of the conical cones measured with five detectors (Edge, SFD, PFD, CC01, and PinPoint chamber). The measurements were shown with and without cross‐calibration at an intermediate field size $3\times 3\;{\text{cm}}^{2}$ for the Edge detector. The percent difference was calculated between OFs measured with different detectors and the data from Varian (downloaded from the Vendor website) measured with the Edge detector

		*6XFFF*							*10XFFF*						
*Cone Size (mm)*		*4*	*5*	*7.5*	*10*	*12.5*	*15*	*17.5*	*4*	*5*	*7.5*	*10*	*12.5*	*15*	*17.5*
OF ‐ the Edge Detector (no cross calibration)		0.607	0.671	0.755	0.800	0.827	0.848	0.859	0.516	0.589	0.700	0.769	0.815	0.847	0.872
OF ‐ the Edge Detector (cross calibration at $3\times 3\;{\text{cm}}^{2}$)		0.608	0.672	0.756	0.801	0.828	0.849	0.860	0.513	0.586	0.696	0.765	0.810	0.842	0.867
% diff. of measured vs. Varian representative OF	Edge	0.8	1.2	0.1	0.8	0.5	0.5	1.0	0.2	0.5	−0.7	0.1	0.0	−0.4	1.3
	SFD	0.3	−1.3	−3.0 ^a^	−2.2 ^a^	−1.9	−1.5	−0.5	1.0	−0.9	−3.7 ^a^	−3.2 ^a^	−2.8 ^a^	−2.7 ^a^	−0.7
	PFD	−7.1 ^a^	−2.6 ^a^	−1.0	0.2	0.2	0.1	0.9	−8.1 ^a^	−3.7 ^a^	−2.2 ^a^	−1.3	−0.9	−1.1	0.6
	CC01	−36.0 ^a^	−24.3 ^a^	−9.5 ^a^	−4.8 ^a^	−2.9 ^a^	−1.7	−0.1	−34.1 ^a^	−23.9 ^a^	−11.6 ^a^	−7.2 ^a^	−4.9 ^a^	−3.7 ^a^	−1.0
	PinPoint	−43.6 ^a^	−32.1 ^a^	−14.3 ^a^	−7.0 ^a^	−4.0 ^a^	−2.6 ^a^	−0.7	−42.7 ^a^	−32.3 ^a^	−17.1 ^a^	−10.2 ^a^	−7.0 ^a^	−5.4 ^a^	−2.6 ^a^

a ^a^ The PFD, CC01, and PinPoint ion chambers show much lower OFs for the smaller cones due to the volume averaging effect.

### C. Couch commissioning

The PRO accuracy (digital reading provided by the linac) at each axis agreed with the measurements within 0.1% with and without weight on the couch. Only 0.1° deviation was observed in the pitch direction with the phantom on the couch. Table 7 summarizes the BB offsets from the isocenter from MV/KV portal image verification. The maximum deviation was 0.5 mm when both pitch and roll were at −3°. For the rigidity test, with both pitch and roll at ± 3°, when the volunteer was off‐centered as much as possible (weight shift), the deviation between the PRO and measurement was 0.1°(3°±0.1°). When the couch was moved laterally to the maximum range, the roll angle deviation became 0.4°. This 0.4° deviation was not due to the rigidity of the couch insert, but due to the rigidity of the upper couch moving mechanism.^(27)^ When the lateral movement of the couch was half of the maximum range, the deviation was 0.2°. The deviation was linear with the lateral offset.

**Figure 9:**
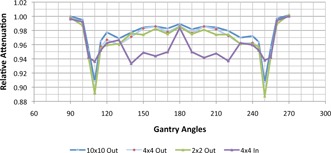
The relative attenuation for the KVue couch at various gantry angles ranging from 90° to 270° using 6X FFF beam at three different field sizes. Relative attenuation is greatest in a small window of oblique entry. The attenuation in positioning of the rails in ‘out’ and ‘in’ positions was studied using a $4\times 4\;{\text{cm}}^{2}$ field size.

Figure 9 shows the relative attenuation of the couch at various gantry angles, ranging from 90° to 270°, using the 6X FFF beam for three field sizes. The attenuation in positioning of the rails in ‘out’ and ‘in’ positions was studied using a $4\times 4\;{\text{cm}}^{2}$ field size. There was a slight decrease in attenuation versus field size. The attenuation properties of KVue imaging couchtop were very similar to the Calypso‐compatible insert. In fact, the CT data and attenuation data were virtually indistinguishable between the two couchtop inserts, so the same couch model can be used in the TPS for both inserts.

**Table 7:** The distance between the BB center and the isocenter after couch pitch and roll positioning

	*Distance (mm)*							
	*No Weight*				*With Weight (13.8 kg)*			
*Pitch/Roll*	*MV AP*		*KV RT Lat*		*MV AP*		*KV RT Lat*	
$0^{{}^{\circ}}/0^{{}^{\circ}}$	0.0	0.0	0.0	L 0.3	S 0.1	0.0	S 0.1	0.0
$+3^{{}^{\circ}}/+3^{{}^{\circ}}$	I 0.2	L 0.2	0.0	L 0.4	0.0	L 0.4	I 0.2	0.0
$-3^{{}^{\circ}}/-3^{{}^{\circ}}$	S 0.1	R 0.1	0.0	0.0	S 0.3	R 0.3	S 0.5	0.0
$0^{{}^{\circ}}/0^{{}^{\circ}}$	0.0	0.0	0.0	L 0.2	S 0.1	0.0	S 0.1	L 0.2

I=inferior; S=superior; L=left; R=right; I 0.2=the BB was 0.2 mm inferiorly from the isocenter.

### D. IMRT and RapidArc commissioning

**Figure 10:**
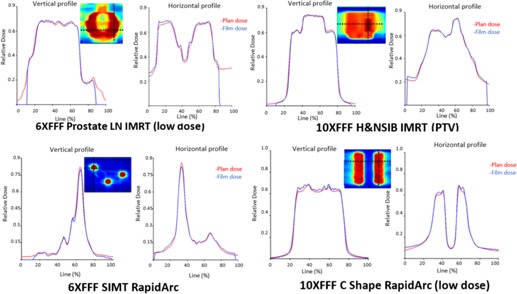
Gafchromic film measurement results for the vertical and horizontal profile comparing the planned versus measured dose in the high‐dose and low‐dose region for both IMRT and RapidArc plans. The red line indicates planned dose, whereas the blue line indicates the measured dose profile. The x‐axis represents the relative position of the selected profile and the y‐axis presents the relative dose.

Composite Gafchromic film and ion chamber results are shown in Table 8 for the measurements in the high‐dose and low‐dose region for both^1^ IMRT and RapidArc plans. The dose difference ratio was −0.0%±1.4% (range, −1.8%−3.5%) for 6X FFF and −0.6%±1.6% (range, −0.5%−4.7%) for 10X FFF in the high‐dose region, and −0.3%±2.3% (range, −4.2%−2.9%) for 6X FFF and 1.5%±3.7% (range, −1.9%−11.9%) for 10X FFF in the low‐dose region. The percentage of points passing the gamma 3%/3 mm criteria for both IMRT and RapidArc plans was 95.5±4.2 (6X FFF) and 97.9±2.7 (10X FFF) in the high‐dose area, and 95.5±3.9 (6X FFF) and 97.5±2.5 (10X FFF) in the low‐dose region. The profiles in the vertical and horizontal directions were analyzed for all tests. Figure 10 shows the analysis of four representative cases.

Table 2 shows the ion chamber and Gafchromic film results for 12 patient SRS/SBRT plans. The average point dose difference was 1.3%±0.9% (range, −2.2%−2.6%) over all the plans. The average gamma pass rate was 99.4%±0.8% for 3%/3 mm criteria and 93.0%±6.0% for 3%/1 mm criteria.

**Table 8:** Composite Gafchromic film and ion chamber results for the measurements in the high‐dose and low‐dose region for both IMRT and RapidArc plans

	*6XFFF*		*10X FFF*	
*Plan*	*Global Gamma* 3%/3 mm	*Point Dose (Percent Difference)*	*Global Gamma* 3%/3 mm	*Point Dose (Percent Difference)*
Hard C IMRT (PTV)	90.6	3.5%	90.0	4.7%
Hard C IMRT (low dose)	87.4	2.4%	91.1	11.9%
Hard C RA (PTV)	93.0	−0.5%	97.5	−0.1%
Hard C RA (low dose)	95.2	−4.2%	98.7	2.4%
HN IMRT (PTV)	94.1	1.0%	98.1	2.5%
HN IMRT (low dose)	97.0	0.4%	99.5	1.3%
HN RA (PTV)	97.9	0.4%	98.7	−0.4%
HN RA (low dose)	98.1	0.2%	98.4	−0.2%
HN SIB IMRT (PTV)	97.5	−0.9%	98.9	1.0%
HN SIB IMRT (low dose)	98.6	−0.2%	97.5	1.7%
HN SIB RA (PTV)	99.0	−0.4%	98.1	−0.7%
HN SIB RA (low dose)	98.1	0.7%	97.8	0.5%
Prostate IMRT (PTV)	95.7	−1.8%	98.2	−0.1%
Prostate IMRT (low dose)	89.6	−3.1%	95.3	−0.2%
Prostate RA (PTV)	99.1	−1.0%	99.3	0.0%
Prostate RA (low dose)	95.7	−3.1%	99.4	−1.9%
Prostate LN IMRT (PTV)	86.2	−0.9%	99.0	−0.5%
Prostate LN IMRT (low dose)	96.2	−0.8%	98.9	−0.2%
Prostate LN RA (PTV)	98.7	0.6%	99.2	0.4%
Prostate LN RA (low dose)	99.0	1.7%	97.9	1.6%
SIMT RA (low dose)	98.9	2.9%	100.0	−0.7%

### E. End‐to‐end testing

**Figure 11:**
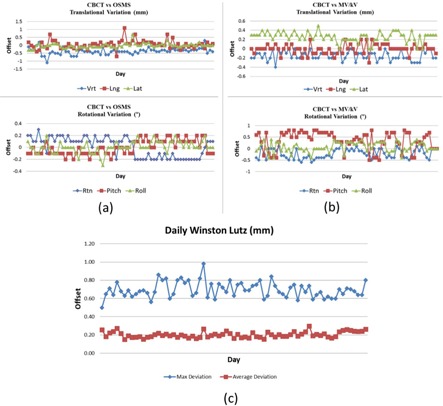
End‐to‐end testing using the OSMS QA phantom from the first three months of operation. The daily variations of isocentric coincidence in the translational and rotational direction between the CBCT and OSMS (a) and between the CBCT and MV/kV planar images (b). The average and maximum absolute values of the daily Winston‐Lutz test performed at four gantry (0°, 90°, 180°, 270°), four couch (0°, 45°, 270°, 315°), and four collimator angles (0°, 45°, 270°, 315°) are shown in (c).

The coincidence of the OSMS and CBCT isocenters was checked on a daily basis. Figure 11(a) shows the daily variations in the translational and rotational direction from the first three months of operation. The daily isocentric coincidence of the CBCT and MV/kV planar imagers is shown in Fig. 11(b). The systematic deviation between the OSMS and CBCT was −0.4±0.2 mm, 0.1±0.3 mm, and 0.0±0.1 mm in the vertical, longitudinal, and lateral directions. There was no residual error in the angular directions. The analysis also showed 0 mm discrepancy in the translational directions between the CBCT and MV/kV orthogonal pair, although 0.1°–0.2° difference was shown in the angular directions. The average and maximum absolute values of the daily Winston‐Lutz test are shown in Fig. 11(c). The mean values and standard deviations of the average deviation and maximum deviation are 0.20±0.03 mm and 0.66±0.18 mm, respectively. The deviations were consistent and within the tolerance (0.75 mm average and 1.0 mm maximum) recommended from TG 142 and the ASTRO quality and safety guidelines for SRS/SBRT.^33^, ^36^

Commissioning was independently verified with the IROC spine and lung credentialing phantoms. All phantoms passed the IROC credentialing; results are shown in Table 9

**Table 9:** Summary of IROC phantom irradiation results for the lung and spine phantoms. The dosimetric precision of TLD is 3%

*Phantom*	*TLD Location*	*IROC vs. Inst*	*Criteria*	*Film Plane*	*Gamma Index*	*Criteria*
Lung Phantom	PTV TLD sup	0.97	0.92–1.08	Axial	100%	≥80%
	PTV TLD inf	0.98	0.92–1.08	Coronal	100%	≥80%
				Sagittal	100%	≥80%
Spine Phantom	PTV TLD sup ant	1.01	0.93–1.07	Axial	90%	≥85%
	PTV TLD inf ant	1.00	0.93–1.07			
	PTV TLD sup post	1.00	0.93–1.07	Sagittal	91%	≥85%
	PTV TLD inf post	0.99	0.93–1.07	

### F. Treatment head leakage test

Figure 3 shows the deep dose equivalent (DDE) map of photon and neutron combined (b), photon only (c) and fast neutron only (d). Thermal neutron dose was within the minimally detectable region of the dosimeters. The maximum measured head leakage dose was 8.45, 6.85, and 1.55 mSv respectively, all located at point E, 0.5 m toward the couch direction. The head leakage from the linac was within 0.1% of the dose at isocenter.

### G. Developer mode

Many iterations of the couch top measurements were required to fully sample the rails and oblique incidence through the couch for different energies (6X FFF and 10X FFF), field sizes ($2\times 2\;{\text{cm}}^{2}$, $4\times 4\;{\text{cm}}^{2}$, and $10\times 10\;{\text{cm}}^{2}$), and shifts in isocenter position (shifts of various magnitude in each of the three translational directions). Automated measurements required only one physicist, while manual measurements required at least two physicists to handle linac positions/beams and data recording. MLC apertures were generated outside of the TPS, and with the .xml file format, double‐checking without use of TPS/operator console was possible. For automated couch top measurements, the time required for each set of angles was approximately 8 min. Without scripting, each set required approximately 11 min. Similar time efficiency gains (approximately 25%) were found for isocenter verification measurements.

## IV. DISCUSSION

This study summarizes the commissioning process of the Edge, a dedicated system for SRS/SBRT treatment. Although it offers the advanced imaging package, the 6DoF treatment couch, and intracranial radiosurgery accessory package, the beam data characteristics and mechanical parameters of the Edge are similar to the TrueBeam.

Beam data from five TrueBeam linacs at three different institutions were previously compared,^(9)^ and we noted excellent agreement between the beam data collected on the Edge and that on the TrueBeam linacs. The CC04 chamber was used to scan the PDDs and profiles for the Edge, while the CC 13 chamber was used for the TrueBeam, and due to its smaller active volume, dose falloff in profiles for the Edge was slightly sharper than that for the TrueBeam. Kim et al.^(37)^ compared PDD and cross‐plane profiles of a 6 MV SRS beam using four different detectors (SFD, PFD, CC01, and CC13). They showed that PDDs from all detectors were in good agreement for field sizes ranging from 1×1 to $6\times 6\;{\text{cm}}^{2}$. Diodes overestimated the dose for field sizes larger than $6\times 6\;{\text{cm}}^{2}$ due to lower energy scattered photons. For profile scans, CC13 ion chamber showed a larger blurring of penumbra even for field size of $10\times 10\;{\text{cm}}^{2}$. A small sensitive volume detector is recommended to achieve a sharper penumbra. However, CC01 (steel electrode) or diode are likely to measure higher dose in the tails due to the overresponse to low‐energy scattered photons.

The dose per pulse at the central axis is higher than off‐axis due to the absence of the flattening filter. Since ion collection efficiency is a function of the dose per pulse, $P_{\text{ion}}$ was measured and compared between the central axis and off axis for two different field sizes (1.007, 1.009, 1.010 at central axis, 2.4 cm off axis and 5.6 cm off axis, respectively, for 6X FFF and 1.011, 1.010, 1.009 for 10X FFF). The consistency of $P_{\text{ion}}$ at different locations ensures there is no additional correction needed for the profile measurement.

Several challenges in small field dosimetry exist, including lack of charged particle equilibrium (CPE), overestimation of field size, perturbation of the particle fluence in the chamber, and volume averaging effect of the detector.^(38)^ Therefore, it is crucial to choose the correct detector, considering the size, energy dependence, and perturbation, for example. A new formalism has been developed for the dosimetry of small field.^(39)^ For the Edge commissioning, the machine‐specific reference field is defined at $3\times 3\;{\text{cm}}^{2}$, since the conventional $10\times 10\;{\text{cm}}^{2}$ cannot be established for all detectors considering the energy dependence of the diodes and volume averaging effect of the ion chambers. The field factor $\Omega_{Q_{cline,}Q_{msr}}^{f_{clin},f_{msr}}$, under the notion proposed by Alfonso et al.,^(39)^ which converts the absorbed dose to water for the machine‐specific reference field ($3\times 3\;{\text{cm}}^{2}$) to the absorbed dose to water for the small clinical field, should be carefully evaluated to account for the difference of the detector response and beam quality at two different field sizes. A Monte Carlo calculated actor $\Omega_{Q_{cline,}Q_{msr}}^{f_{clin},f_{msr}}$ was recommended to correct the field factor. Several studies have been published since then to generate correction factors for various detectors from different treatment platforms.^40^, ^41^ The diodes were shown to have an overresponse at small fields. A correction factor should be applied to the SFD for field sizes less than $1\times 1\;{\text{cm}}^{2}$ and the Edge detector for field sizes within $1.5\times 1.5\;{\text{cm}}^{2}$.^(41)^ This factor might also explain the 4% difference in the output factor measurements between the Edge detector and SFD for conical cones. A Monte Carlo simulation for the FFF beams may be beneficial in verifying the correction factors for stereotactic diodes at very small field sizes (<2 cm).

There are various methods to measure the DLG: 1) measuring the distance between the radiation and geometrical field edge of a MLC‐defined field size; 2) matching the gap width profiles with the measured values; 3) optimizing the parameters based on treatment delivery; and 4) sweeping MLC leaves with a variety of sliding MLC gap widths.^42^, ^43^ For the Eclipse TPS, only one DLG value can be commissioned for all different field sizes and delivery techniques. Therefore, there is a trade‐off in the optimal DLG between IMRT and RapidArc measured fields, as well as the fields with different sizes and modulation. The difference between the measured and optimized DLG values is caused by different contributions to the dose from the beam penumbra, which is a consequence of different patterns of leaf movement. Szpala et al.^(44)^ found out that the DLG values are a function of the distance (in the BEV) between the dose point and the leaf ending, and the width of the MLC slit. Therefore, calculation using a single DLG value may overestimate the measurement in the proximal penumbra, while it may underestimate the dose in the distal penumbra for RapidArc delivery.^(44)^ For IMRT delivery, the DLG values for smaller and larger regions average out and a single value can serve as the optimal value for different widths of the MLC slits.^(44)^ Therefore, the DLG values were optimized for RapidArc delivery by evaluating the measured and calculated dose for selected spine radiosurgery cases due to the requirement of an extremely steep dose gradient. The adjustments did not have much impact on the IMRT delivery. The dose calculation accuracy was further validated in a more comprehensive manner, using test cases representative of various clinical treatment sites.

Tissue maximum ratios (TMR) and off axis ratios are used for the cone‐based dose calculation. TMR values can be measured by draining or filling water in a 3D water tank or derived from PDD curves. It is challenging to use the conventional conversion methods, since phantom scatter factors for small fields are difficult to measure. Van Battum et al.^(45)^ proposed to obtain TMR values from PDD curves and total scatter factors. A depth‐dose curve corrected for source detector distance was generated from existing PDD curves and the dose at each depth and field size was fitted by a double exponential function. TMR was then calculated by taking the ratio of the dose at the depth of interest and the reference depth. Battum and colleagues reported the agreement between calculated and measured TMR was within 2%. TMR values were spot checked on the Edge system at nine points for each cone and compared against the converted data. The difference was within 2% except at 20 cm, the deepest depth. Larger discrepancies were noted at depths beyond 20 cm, which is generally greater than the maximum depth required for intracranial SRS treatment. This method can be considered an alternative option to obtain TMR values for cones when a precise TMR measurement is not available from the water tank.

Conical cones may provide a sharper beam penumbra than the MLCs since the cone is closer to the isocenter and more transmission occurs at the round leaf ends of the MLCs. The beam penumbra for the cones is a function of depth, cone size, and energy. It increases as the cone size, depth or energy increases. The beam penumbra increases faster for the 90%−10% value than for the 80%−20% value, as shown in Fig. 8.

The 6DoF (PerfectPitch) couch top is equipped with rails, which will lead to errors in the delivered dose if the rails are not properly accounted for the in treatment plan. This is especially important in the context of spine SRS, where highly modulated posterior beams are used and the isodose fall off from 90% to 50% line is on the order of 3 mm. Therefore, the attenuation effect of the rails and couch tops should be measured. A proper couch model should be established in the TPS according to recommendations from AAPM Task Group Report No. 176.^(46)^ By taking CT scans of the couch top prior to installation on the treatment unit, couch models can be developed, along with a setup for future planning and delivery to a QA phantom. In this study, such a couch model was incorporated for all the test plans related to the Edge commissioning, phantom QA, and patient planning. The couch model is also used for routine patient treatment planning.

To optimize use of the couch model for RapidArc delivery, one solution is to place both couch rails in the ‘in’ position and start the arc at oblique angles to avoid the beam traversing through the rails. However, the rigidity of the couch insert should be carefully evaluated in the lateral direction (patient left and right) for such a configuration. The deviation was linear with lateral translation, due mainly to the rigidity of the couch moving mechanism.

The AAA dose calculation algorithm was reported to overestimate the dose beyond low‐density inhomogeneities.^(47)^ Errors could be greater than 2.5% when using the AAA to calculate the dose when the beams transverse a large air gap from the treatment couch or an immobilization device to the patient.^(48)^ The Acuros XB algorithm, a numerical method based on linear Boltzmann transport equation solver, is also available in the Eclipse TPS. The algorithm has been compared to the AAA in numerous studies, and demonstrated that it has better agreement with full MC simulation in slab phantoms containing various materials.^49^, ^50^ Rana et al.^(51)^ reported the dose calculation using the Acuros XB had better agreement with the measurement than using the AAA in the inhomogeneous phantoms. The validation of the AAA and Acuros XB dose calculation algorithm using a heterogeneous phantom will be performed in the second tier commissioning.

Since target localization may incorporate single or multiple imaging modalities and 6DoF couch correction, end‐to‐end tests were designed to evaluate the coincidence of each imaging modality with the radiation isocenter, the accuracy of 3D–3D and 2D–3D image registration, the precision of 6DoF correction, and the coincidence of gantry, collimator, and couch axes with the radiation isocenter. The laser and crosshair alignment should also be checked after the phantom localization. By performing the Winston‐Lutz test on a daily basis, the localization accuracy can be accessed and deviations can be easily identified to trigger further action, such as imaging system calibration, couch precision test, or linac mechanical check.

## V. CONCLUSIONS

We present technical aspects related to comprehensive commissioning and assessment of localization and delivery accuracy of a novel, linac‐based SRS/SBRT‐based treatment system (The Edge, Varian Medical Systems). We have demonstrated that the beam characteristics and localization accuracy of this system are well suited for the frameless, linac‐based SRS, SBRT treatments, and other general treatment indications in radiation oncology.

## ACKNOWLEDGMENTS

This work has been supported in part by a research grant from Varian Medical Systems, Palo Alto, CA.

## Supporting information

Supplementary Material
