# Supplementary material for: Characteristics of a novel treatment system for linear accelerator–based stereotactic radiosurgery
Source: J Appl Clin Med Phys. 2015 Jul 8;16(4):125–48. doi: 10.1120/jacmp.v16i4.5313 (PMC5690003; doi:10.1120/jacmp.v16i4.5313)
Supplement: Supplementary file 1 — Supplementary Material [file ACM2-16-125-s001.pdf]

Characteristics of ~~the~~ a novel treatment system for Linear Accelerator–based stereotactic radiosurgery

Abstract:

Purpose: The purpose of this study is to characterize the dosimetric properties and accuracy of a novel treatment platform (Edge^{TM} radiosurgery system, Varian Medical Systems, Palo Alto, CA) for localizing and treating patients with frameless, image guided, stereotactic radiosurgery (SRS) and stereotactic body radiotherapy (SBRT).

Methods and Materials: Initial measurements of various components of the system, such as a comprehensive assessment of the dosimetric properties of the 6, 10X flattening filter free (FFF) beams for both high definition (HD120^{TM}) MLC and conical cone based treatment, positioning accuracy and beam attenuation of a six degree of freedom (6DoF) couch, treatment head leakage test and integrated end-to-end accuracy tests, have been performed. The end-to-end test of the system was performed by CT imaging a phantom, and registering hidden-targets on the treatment couch to determine the localization accuracy of the optical surface monitoring system (OSMS), Cone Beam CT (CBCT), and MV imaging systems, as well as the radiation isocenter targeting accuracy.

Results: The deviations between the percent depth dose curves acquired on the new LINAC-based system (Edge), and the previously published machine with FFF beams (~~Truebeam~~TrueBeam) beyond D_{max} were within 1.0% for both energies. The maximum deviation of output factors between the Edge and ~~TrueBeam~~TrueBeam was 1.6%. The optimized dosimetric leaf gap values, which were fitted using Eclipse dose calculations and measurements based on representative spine radiosurgery plans, were 0.700 mm and 1.000 mm respectively. For the conical cones, 6XFFF has sharper penumbra ranging from 1.2 – 1.8 mm (80% - 20%) and 1.9 – 3.8 mm (90% - 10%) relative to 10XFFF, which has 1.2 – 2.2 mm and 2.3 – 5.1 mm respectively.

The relative attenuation measurements of the couch for PA, PA (rails-in), oblique, oblique (rails-out), oblique (rails-in) were: -2.0%, -2.5%, -15.6%, -2.5%, -5.0% for 6XFFF and -1.4%, -1.5%, -12.2%, -2.5%, -5.0% for 10XFFF respectively with a slight decrease in attenuation versus field size.

The systematic deviation between the OSMS and CBCT was -0.4 ± 0.2 mm, 0.1 ± 0.3 mm and 0.0 ± 0.1 mm in the vertical, longitudinal, and lateral directions. The mean values and standard deviations of the average deviation and maximum deviation of the daily Winston-Lutz tests are 0.20 ± 0.03 mm and 0.66 ± 0.18 mm respectively.

Conclusions: Initial testing of this novel system demonstrates the technology to be highly accurate and suitable for frameless, LINAC-based SRS and SBRT treatment.

KEYWORDS: Commissioning, Edge, 6DoF couch, Conical Cones, End-to-End Testing

I. INTRODUCTION

Since the term "stereotactic radiosurgery" was coined by Lars Leksell in 1951, there have been many technological, biological and clinical advances in the field of stereotactic radiosurgery[1-4]. The accuracy of linear accelerators (linacs) has been improved significantly since 1980s[5-7] and LINAC-based radiosurgery has been widely adopted over the subsequent decades. Since the 1990s, various technological advances have taken place to allow very precise treatments. The dedicated LINACs have been designed exclusively for radiosurgery to further improve the targeting accuracy and high dose rate delivery. The mechanical isocenter accuracy of the C-arm LINAC has reached sub millimeter levels [8, 9]. The flattening filter was first redesigned to be more efficient and later completely removed in order to deliver higher dose rates[10, 11]. The multi-leaf collimators (MLC) leaf resolution is also improving, with 2.5 mm leaf widths at the isocenter, in order to improve the dose conformality to the target[12]. Treatment delivery methods have advanced to further improve conformality to complex geometric targets, while limiting dose to critical organs, such as dynamic conformal arc (DCA), IMRT and VMAT[13-16]. In the era of image guidance, numerous methods have been developed for stereotactic treatment delivery, including optical surface monitoring, in-room CT, stereoscopic X-ray imaging, ultrasound and cone beam computed tomography (CBCT)[17-20]. Image guided frameless treatment has been systematically studied and the positioning accuracy has been validated for the use in stereotactic treatments[20, 21].

The latest platform for LINAC-based SRS treatments (the Edge, Varian Medical Systems, Palo Alto, CA) offers multiple imaging modalities for treatment localization, including an optical surface monitoring system (OSMS) for surface tracking, 2.5 MV portal images for verification, automatically triggered monoscopic kV imaging to track intra-fractional motion, 4D CBCT to evaluate tumor motion offline, extended CBCT images by stitching multiple CBCT scans together, and a Calypso/Varian electromagnetic beacon-based tracking system. The new couch (PerfectPitch™) supports six degrees-of-freedom (6DoF) corrections from multiple imaging modalities for precise patient setup. The flat panel imager is designed with a greater dynamic range, faster image readout rate, and a larger active area. This technology also has a stereotactic accessory package which includes conical cones ranging in diameter from 4 to 17.5 mm in diameter. Here we describe a comprehensive commissioning process suitable for modern, LINAC-based SRS/SBRT with focus on the characterization of beam parameters, conical cones, 6DoF couch, dosimetric verification, and integrated end-to-end tests of this new technology.

II. MATERIALS AND METHODS

II.A. Flattening Filter Free (FFF) Beam commissioning

Beam data was measured for the purpose of generating a beam model for the convolution/superposition dose algorithm (Anisotropic Analytical Algorithm, AAA v 11.0.31 within the Eclipse Treatment Planning System (TPS), ~~,~~ Varian Medical Systems, Palo Alto CA). Measurements were performed for the two beam energies configured for our LINAC (flattening filter free photons, 6XFFF and 10XFFF). AAPM task group report No. 45"AAPM Code of Practice for Radiotherapy Accelerators" recommendations were followed for commissioning tasks[22]. Selection of different detectors for water phantom measurements were based on AAPM task group report No. 106 and small field dosimetry specification [23, 24] (Table 1). Field sizes ranged from 1×1 cm^2 to 40 × 40 cm^2 which were determined by the jaw (i.e. data was acquired with the MLCs parked). All mandatory and recommended beam data measurements (PDDs, cross-plane and in-plane profiles) were performed, as specified in the Eclipse manual for commissioning the AAA algorithm beam model.

II.A.1. Percent Depth Dose and Profiles

PDDs and profiles were scanned for ~~fourteen~~ ten different field sizes, ranging from 1 × 1 to 40 × 40 cm^2 at an SSD of 100 cm. The central electrode of the chamber was oriented parallel to the in-plane direction, perpendicular to the beam axis.~~ As specified by the Eclipse manual, ~~ ~~t~~The effective point of measurement correction was applied during the beam scanning since ~~PDD data were not shifted to correct for effective point dose measurement, since~~ the AAA does not perform this correction~~ is~~ automatically~~ applied in the TPS during the beam modeling process~~. Cross-plane and in-plane profiles were acquired at five different depths (d_{max}, 5, 10, 20 and 30 cm) for each field size. PDD and profiles curves were measured with a CC04 cylindrical chamber (Scanditronix Wellhofer, IBA Dosimetry America, Barlett, TN, USA) for field sizes equal or greater than 2 × 2 cm^2 using the 400 MU/min dose rate. The SFD (Scanditronix Wellhofer, IBA Dosimetry America, Barlett, TN, USA) was used for field sizes 1 × 1 cm^2 and 2 × 2 cm^2. These curves were used for our own small field dosimetry evaluation since the profile or PDD curves for field sizes smaller than 2 x 2 cm^2 are not used by the beam configuration in Eclipse [25]. A reference detector was not used for the diode measurement. Data was acquired with the field detector in a step-by-step mode, with data sampled at every 0.3~~2~~ mm. The beams were scanned at the maximum dose rate and the acquisition sampling was set to improve the signal to noise ~~ratio[24]~~ratio [23]. Both PDD and profile curves were compared to data acquired from

other LINACs in our clinic with FFF beam configurations (~~TrueBeam~~TrueBeam linacs, Varian Medical Systems, Palo Alto CA)[9].

The linearity response with dose rate of the CC04 chamber was measured for 6XFFF (range: 400 – 1400 MU/min) and 10XFFF (range: 400 – 2400 MU/min) with a fixed MU. The ion chamber collection efficiency was also measured for both energies at the maximum dose rate for field sizes of 10 × 10 and 15 × 15 cm^2. The two-voltage method (300V and 150V) was used to calculate the recombination correction factor (P_{ion}) at the central axis and one off-axis position (2.4 and 5.6 cm off-axis, transverse plane) for each field size.

II.A.2. Output Factors (OFs)

Total scatter factors (S_{cp}) were acquired at 95 cm SSD and 5 cm depth using a CC04 ion chamber at field sizes ranging from 3 × 3 to 40 × 40 cm^2. The SFD was used for field sizes from 1 × 1 to 3 × 3 cm^2. The diode was cross calibrated with the CC04 at 3 × 3 cm^2 as follows:

$$\frac{SFD(fs)}{SFD(3\times 3)}\times\frac{CC04(3\times 3)}{CC04(10\times 10)} \qquad (1)$$

where SFD (*fs*) is the diode reading for the small field size, SFD (3×3) is the diode reading for the 3 × 3 cm^2 field, CC04 (3×3) is the reading of the CC04 chamber for the 3 × 3 cm^2 field, and CC04 (10×10) is the reading of the CC04 chamber for the field size 10x10 cm^2.

II.A.3. MLC Leaf Transmission and Dosimetric Leaf Gap (DLG) Measurements

The MLC leaf transmission and DLG were commissioned as follows: The baseline values were measured through extrapolation to a leaf gap of zero on a plot of dose as a function of the gap between opposite leaves[25]. The values were then iteratively adjusted using three representative spine radiosurgery plans (vertebral body, paraspinal mass and spinous process) for the purpose of optimizing agreement between calculations and measurements for both IMRT and RapidArc techniques. Point doses were measured using a PTW pin point chamber 31014 (PTW, Freiburg GmbH, Germany) in a Lucy phantom (Standard Imaging Inc., Middleton, WI). Planar doses were measured using Gafchromic EBT3 films (International Specialty Products, Wayne, NJ) sandwiched at the center of a 10cm thick acrylic phantom (Brainlab, Feldkirchen, Germany).

II.B. Conical Cones Commissioning

The Edge conical collimator accessory system consists of seven circular cones, 4, 5, 7.5, 10, 12.5, 15, 17.5 mm in diameter. The cones are inserted in an accessory mount that attaches to the collimator face plate, with an Integrated Conical Collimator~~-~~ Verification & Interlock (ICVI) system which recognizes a specific cone during mounting and dismounting. PDD data was acquired at SSD of 100 cm using the SFD and converted

to TMR values using the standard conversion method [26]. The off-axis profiles were scanned in both in-plane and cross-plane directions at the depth of 5 cm at three SSDs: 80, 90 and 100 cm. Output factors (OFs) for all cones were measured with a 5 × 5 cm^2 jaw size at 95 cm SSD and 5 cm depth for both 6XFFF and 10XFFF modes using five different detectors (Table 1): Edge diode (Sun Nuclear, Melbourne, FL), SFD, photon diode (Scanditronix Wellhofer, IBA Dosimetry America, Barlett, TN, USA), CC01 chamber, and pinpoint chamber 31014. All the diodes were cross calibrated with the CC04 at the 3 × 3 cm^2 field size. Results were compared with the manufacturer representative data measured with the Edge diode.

II.C. Six-degree-of-freedom (6DoF) Couch Commissioning

Couch commissioning procedures included positioning accuracy of the imaging system and couch to detect linear and rotational offsets, rigidity test of the couch insert in the lateral direction with both rails at the center (‘in’ position), and attenuation measurements of the rails and inserts. ~~, rigidity test of the couch insert in the lateral direction with both rails at the center (‘in’ position), and positioning accuracy of the imaging system and couch to detect linear and rotational offsets.~~

II.C.1. 6DoF Positioning Accuracy

The accuracy of the couch position readout of each of the six axes was validated at various positions with and without a Rando Pelvic phantom (13.8 kg) placed on the couch. The positional read-out (PRO) accuracy was verified at ten positions (±1, ±2, ±5, ±10, ±20 cm) using a tape measure in each translational direction, four positions (45º, 90º, 315º, 270º) using a protractor in the yaw direction, and seven positions (0º, ±1º, ±2º, ±3º) using a digital level in the pitch and roll direction. The pitch and roll positioning uncertainties of the online image registration were evaluated using the OSMS QA phantom (Vision RT, London, UK) with and without the Rando phantom to evaluate the weight factor. The central BB in the phantom was aligned to the isocenter using MV/KV orthogonal pair imaging. A given pitch and roll were applied (+3°/+3°, -3°/-3°, and 0°/0°), a MV/KV image pair was taken, and the distance between the center of the BB and isocenter was measured to evaluate the pitch and roll positioning accuracy.

II.C.2. Rigidity Test of Couch Insert

The rigidity test was performed at two couch positions in the longitudinal direction with a volunteer (96.2 kg) lying on the couch. The volunteer was positioned at the center of the Calypso-compatible couchtop insert and the couch was also centered laterally. A three degree pitch and roll was applied to the couch. The pitch angle was given to evaluate the potential influence on the roll. A digital level was used to check for possible angular deviation at the longitudinal end of the couch insert. The couch rigidity in roll angle with respect to

the couch position in the lateral direction was also tested by off-centering the volunteer to the maximum lateral direction at 24.8 cm[27].

II.C.3. Beam Attenuation through the Couch Top and Rails

The couch top consists of two mobile, kevlar support rails, a nonconductive Kevlar Varian/Calypso insert, and a solid carbon fiber KVue insert. Prior to installation of the linac, both Calypso and KVue inserts along with the support rails were CT scanned with the rails at various positions. An additional scan with the couch top 15 cm above the CT table top was obtained with 20 cm solid water to mimic patient-like setups. The attenuation measurements were obtained for field sizes of 2, 4 and 10 cm^2 at 42 gantry angles including six pairs of opposing fields and other oblique angles in which the beams traversed the couch inserts and/or rails. The results were then used to determine an accurate structure model for the planning system.

II.D. IMRT and RapidArc Commissioning

A total of 21 plans generated using updated AAPM TG 119 test suite [28] were planned and calculated with the AAA, V.11.0.31 algorithm in the Eclipse TPS. A solid water phantom (density: 1.03 g/cm^3) was used to evaluate the dosimetric accuracy of both energies using the maximum dose rate. The actual dose rate varied during the delivery for the RapidArc plans. The 6DoF couch top with the rails in the 'out' position was included in the dose calculation. The 21 treatment plans included hard C shape, head & neck, head & neck with simultaneous integrated boost, prostate, prostate and lymph nodes, and single isocenter multiple intracranical targets (SIMT) (Figure 1). All IMRT cases used 7-9 beams and RapidArc cases used 2 arcs, except for the SIMT case, which used 4 arcs, with dose optimization constraints that follow Clark et al.'s technique[29]. Point dose measurement using an Both ion chamber (PTW PinPoint Chamber, Model 31014) and planar dose distribution measurement using films (Gafchromic EBT3) measurements were performed in both the high dose target and a low dose (OAR) region. For the SIMT case, the distance between the isocenter and the center of each of three targets was 2, 4 and 4.5 cm respectively and 16 Gy was delivered to each target. Ion chamber measurements were made at the isocenter and the center of one of the targets 2 cm away. Film was delivered in the axial plane 1 cm posterior to the isocenter. An in-house software was developed to integrate Gafchromic film dosimetry protocol using EBT3 films which streamlines a dose pattern delivery for calibration, calibration curve fitting, film scanning in the fixed scanner position, dose mapping from multiple color channels, and profile/gamma analysis[30].

II.E. The End-to-End Test~~s~~

Daily end-to-end quality assurance tests were performed to assess the overall accuracy of the system from CT simulation, treatment planning, image based localization and final treatment delivery using the OSMS QA phantom. The phantom is a polystyrene 15 × 15 × 15 cm^3 cube embedded with five 7.5 mm diameter ceramic BBs (Figure 2(a)). One of the BBs was located at the center of the cube. The phantom was scanned with 0.8 mm slice thickness (pixel size 0.6 × 0.6 mm^2) without the base plate. The cube and BBs were contoured in Eclipse and used as the reference image. In the treatment room, the phantom was setup on top of an acrylic base plate and fixed to the pegs of an indexing bar for consistent setup. The acrylic plate was engraved with three notches in which the three screws of the OSMS phantom holder were seated. The couch was set at a fixed position (vertical: 10.0cm; longitudinal: 98.5cm; lateral: 0.0cm, pitch: 0.5° and roll: 0.5°). The OSMS system was first used to localize the phantom surface and the difference (delta) between the current position of the OSMS phantom and its reference position was recorded (Figure 2(b)). CBCT images of the phantom (kV=100; mAs=265, 1 mm slice thickness, full fan) were acquired and automatic fusion was performed after adjusting the contrast of the acquired image and reference image to achieve optimal window and leveling in order to visualize the BBs (Figure 2(c)). 6D fusion shifts were recorded and applied. The phantom position in the OSMS system after correction was recorded to evaluate the residual error. An orthogonal MV/KV set was taken and 2D-3D image fusion was performed to quantify the residual error (Figure 2(d & e)). An Electronic Portal Imaging ~~Dosimetry~~ Device (EPID)-based Winston Lutz (WL) test was then performed to verify the isocenter targeting accuracy. Twelve 2 × 2 cm^2, MLC-defined portal images were acquired at four gantry, four couch and four collimator angles, which were analyzed by an in-house developed C++ software based on an open-source framework (Insight Segmentation and Registration Toolkit 4.3.2) to measure the distance between the center of the central BB and the full width at half maximum (FWHM) of the radiation field (Figure 2(f)). The coincidence of the imaging systems and radiation isocenter are evaluated on a daily basis according to AAPM TG 142 recommendation [31].

Independent end-to-end tests were performed using the Imaging and Radiation Oncology Core (IROC-Houston) spine and thorax phantoms. The phantoms were scanned, treatment planned and irradiated at our institution according to the IROC-Houston credentialing criteria. After irradiation, the phantoms were sent back to IROC-Houston, where absolute point dose was measured with TLDs and 2D film dose planes were measured with Gafchromic EBT2 film, analysis was completed independently by IROC-Houston. Treatment plans were generated with the Eclipse TPS using the same AAA algorithm and delivered using the RapidArc

technique for the spine phantom and IMRT for the thorax phantom. Both phantoms were localized using the OBI system, where CBCT was used for initial set up.

The spine phantom consists of a pentagon shaped PTV (42 cc) abutting bone and a cylindrical spinal cord structure, the PTV is set between the right and left lung structures. The spine phantom has four TLDs within the PTV structure in the high dose region and one within the heart in the low dose region. Two films bisect the PTV in the axial and sagittal planes. The thorax phantom consists of an ellipsoidal shaped PTV (72 cc) located in the middle of a cylindrical volume of lung. The thorax phantom contains two TLDs within the PTV, and two TLDs in the low dose region, one in the heart and one in the cord. Three films bisect the PTV in the axial, coronal, and sagittal planes.

~~Independent end-to-end tests were performed in using the Imaging and Radiation Oncology Core (IROC-Houston) spine phantom. Treatment plans were generated and delivered to the spine phantom using RapidArc and to a thorax phantom using IMRT. The plans were planned and delivered according to the IROC-Houston instructions and the phantoms were localized using CBCT. The point dose and 2D dose planes were analyzed by IROC-Houston.~~

II.F. Treatment Head Leakage Test

Treatment head leakage was measured using 30 pairs of Luxel+ T series dosimeters (Landauder, Glenwood, IL) placed around a 2 meter radius circular plane, in a plane perpendicular to the beam axis at the isocenter. Figure 3(a) shows the placement of each pair of dosimeters. 10,000 MU were delivered to the dosimeters at gantry 0° position with both MLC and jaw at most closed position using the highest energy, 10XFFF, at 2400 MU/min. The average reading of each pair of dosimeters was recorded.

II.G. Developer Mode

The Edge system includes Developer Mode enabling the use of XML-scripting for automation of commissioning and QA procedures. XML-scripting was used for various commissioning tasks including beam scanning, couch modeling, and end-to-end tests.

III. Results

III. A. Beam Commissioning

III.A.1. Percent Depth Dose and Profile Evaluation

Figure 4 shows the PDD curves normalized at Dmax for 6XFFF (a) and 10XFFF (b) for the field sizes ranging from 1 × 1 to 40 × 40 cm^2. Table 2 summarizes the D_{max} and PDD values at 5, 10, 20, and 30 cm depth. The deviations between the photon beam curves acquired on the new LINAC-based system (Edge), and the

previously published machine with FFF beams (~~Truebeam~~TrueBeam) beyond D_{max} were within 1.0% for both energies. The beam quality specifier (%$dd(10)_x$) for the Edge was 63.0% and 70.6~~5~~% for 6XFFF and 10XFFF respectively without 1 mm lead foil. With a 1mm lead foil, %$dd(10)_x$ increased to 71.1% for 10XFFF, however, the difference between the quality conversion factors (k_Q) for 10XFFF were within 0.1% with and without the lead foil.

Figures 4 (c & d) illustrate the cross-plane profiles measured at 10 cm depth for all 10~~5~~ field sizes from 1 × 1 to 40 × 40 cm^2. The curves are normalized to 100% on the central axis. Since only FFF modes were commissioned for the Edge, we could not use the penumbra normalization method proposed by Pönisch et al[32]. Figure 5 shows direct comparison of profile curves between the Edge and the ~~TrueBeam~~TrueBeam for two representative fields using 10XFFF: 2 × 2 cm^2 and 10 × 10 cm^2. The profiles between the Edge and the ~~TrueBeam~~TrueBeam were practically the same with slightly sharper penumbra obtained on the Edge at all the depths.

The values of P_{ion} at the central axis and two off-axis positions were compared. The output constancy was within 0.1% with various dose rates for both energies. The ion chamber collection efficiency off-axis agreed within 0.3% of the values at the central axis for the two field sizes evaluated.

III.A.2. Output Factors

The output factors S_{cp} for the symmetrical fields and rectangular fields are tabulated in Table 3 and 4 for 6XFFF and 10XFFF, respectively. The shielded area in the table corresponds to data measured with the SFD detector. S_{cp} for symmetrical fields ranging from 1 × 1 to 40 × 40 cm^2 were also plotted in Figure 6 and compared against the ~~TrueBeam~~TrueBeam machine (Figure 6(b)). The maximum deviation between the Edge and ~~TrueBeam~~TrueBeam was 1.6% for field size of 1 × 2 cm^2 (6XFFF) and 1.0% for 1 × 1 cm^2 (10XFFF).

III.A.3. HDMLC Transmission and DLG

The measured DLG values were 0.507 mm for 6XFFF and 0.622 mm for 10XFFF. Optimized values, which were fitted using Eclipse dose calculations and measurements based on representative spine radiosurgery plans, were 0.700 mm and 1.000 mm respectively. The MLC transmission values were 1.209% for 6XFFF and 1.427% for 10XFFF. Dose difference ratios of ion chamber measurements were 0.015% ± 0.008% for 6XFFF and 0.010% ± 0.010% for 10XFFF and the passing rates for 2%/2 mm gamma criteria were 98.0 ± 1.0 for 6XFFF and 96.9 ± 1.9 for 10XFFF after the DLG optimization.

III.B. Conical Cones

Figure 7 (a & b) shows the PDD data for the conical cones for 6XFFF and 10XFFF. The ~~in-plane and cross-plane profile data~~off-axis ratios for all the conical cones at the depth of 5 cm at 100 cm SSD are shown in Figure 7 (c & d). All beam profile data were normalized to the central axis. The beam penumbra (width between 90% - 10% and 80% - 20%) increases as the diameter of the cone increases as shown in Figure 8. 6XFFF has sharper penumbra ranging from 1.2 – 1.8 mm (80% - 20%) and 1.9 – 3.8 mm (90% - 10%) relative to 10XFFF, which has 1.2 – 2.2 mm and 2.3 – 5.1 mm respectively.

Table 5 shows the OFs of the cones using the Edge detector with and without cross calibration at an intermediate field size. Because the Edge detector is independent of variation in energy spectrum[33], minimal difference between the two measurements was observed (OFs were within 0.2 and 0.7% for 6XFFF and 10XFFF, respectively).

The percent difference between OFs we measured using different detectors and the data from the manufacturer measured with the Edge detector (available at the Vendor website) is also shown in Table 5. The difference was ~1% for the Edge detector and increased to 4% for the SFD detector. As observed in Table 5, the PFD, CC01 and Pinpoint ion chambers show much lower OFs for the smaller cones due to the volume averaging effect.

III.C. Couch Commissioning

The PRO accuracy (digital reading provided by the Linac) at each axis agreed with the measurements within 0.1% with and without weight on the couch. Only 0.1° deviation was observed in the pitch direction with the phantom on the couch. Table 6 summarizes the BB offsets from the isocenter from MV/KV portal image verification. The maximum deviation was 0.5 mm when both pitch and roll were at -3°. For the rigidity test, with both pitch and roll at ±3°, when the volunteer was off-centered as much as possible (weight shift), the deviation between the PRO and measurement was 0.1° (3° ± 0.1°). When the couch was moved laterally to the maximum range, the roll angle deviation became 0.4°. This 0.4° deviation was not due to the rigidity of the couch insert, but due to the rigidity of the upper couch moving mechanism[27]. When the lateral movement of the couch was half of the maximum range, the deviation was 0.2°. The deviation was linear with the lateral offset.

Figure 9 shows the relative attenuation of the couch at various gantry angles ranging from 90° to 270° using the 6XFFF beam for three field sizes. The attenuation in positioning of the rails in ‘out’ and ‘in’ positions was studied using a 4 × 4 cm^2 field size. There was ~~relative attenuation measurements for PA, PA (rails-in),~~

~~oblique, oblique (rails-out), oblique (rails-in) were: -2.0%, -2.5%, -15.6%, -2.5%, -5.0% for 6X FFF and -1.4%, -1.5%, -12.2%, -2.5%, -5.0% for 10X FFF, respectively with~~ a slight decrease in attenuation versus field size. The attenuation properties of KVue imaging couchtop were very similar to the Calypso-compatible insert. In fact, the CT data and attenuation data was virtually indistinguishable between the two couchtop inserts, so the same couch model can be used in the TPS for both inserts.

III.D. IMRT and RapidArc Commissioning

Composite Gafchromic film and ion chamber results are shown in table 7 for the measurements in the high-dose and low-dose region for both IMRT and RapidArc plans. The dose difference ratio was -0.0% ± 1.4% (range, -1.8% – 3.5%) for 6XFFF and -0.6% ± 1.6% (range, -0.5% – 4.7%) for 10XFFF in the high-dose region and -0.3% ± 2.3% (range, -4.2% – 2.9%) for 6XFFF and 1.5% ± 3.7% (range, -1.9% – 11.9%) for 10XFFF in the low-dose region. The percentage of points passing the gamma 3%/3 mm criteria for both IMRT and RapidArc plans was 95.5±4.2 (6XFFF) and 97.9±2.7 (10XFFF) in the high-dose area and 95.5±3.9 (6XFFF) and 97.5±2.5 (10XFFF) in the low-dose region. The profiles in the vertical and horizontal directions were analyzed for all tests. Figure 10 shows the analysis of four representative cases.

III. E. End-to-End Testing

The coincidence of the OSMS and CBCT isocenters was checked on a daily basis. Figure 11 (a~~ & b~~) shows the daily variations in the translational and rotational direction from the first three months of operation. The daily isocentric coincidence of the CBCT and MV/kV planar imagers is shown in Figure 11 (~~c & d~~b). The systematic deviation between the OSMS and CBCT was -0.4 ± 0.2 mm, 0.1 ± 0.3 mm and 0.0 ± 0.1 mm in the vertical, longitudinal, and lateral directions. There was no residual error in the angular directions. The analysis also showed 0 mm discrepancy in the translational directions between the CBCT and MV/k~~K~~V orthogonal pair, although 0.1-0.2° difference was shown in the angular directions. The average and maximum absolute values of the daily Winston-Lutz test are shown in Figure 11 (c~~e and f~~). The mean values and standard deviations of the average deviation and maximum deviation are 0.20 ± 0.03 mm and 0.66 ± 0.18 mm respectively. The deviations were consistent and within the tolerance (0.75 mm average and 1.0 mm maximum) recommended from TG 142 and the ASTRO ~~-~~Quality and safety guidelines for SRS/SBRT[31, 34]. Commissioning was independently verified with the IROC spine and lung credentialing phantoms. All phantoms passed the IROC credentialing; results are shown in table 8.

III.F. Treatment Head Leakage Test

Figure 3~~2~~ shows the deep dose equivalent (DDE) map of photon and neutron combined (b), photon only (c) and fast neutron only (d). Thermal neutron dose was within the minimally detectable region of the dosimeters. The maximum measured head leakage dose was 8.45, 6.85 and 1.55 m~~rem~~Sv respectively, all located at point E, 0.5 m toward the couch direction. The head leakage from the linac was within 0.1% of the dose at isocenter.

III.G. Developer Mode

Many iterations of the couch top measurements were required to fully sample the rails and oblique incidence through the couch for different energies (6XFFF and 10XFFF), field sizes (2 × 2 cm^2, 4 × 4 cm^2, and 10 × 10 cm^2), and shifts in isocenter position (shifts of various magnitude in each of the three translational directions). Automated measurements required only one physicist, while manual measurements required at least two physicists to handle LINAC positions/beams and data recording. MLC apertures were generated outside of the TPS, and with the .xml file format, double-checking without use of TPS/operator console was possible. For automated couch top measurements, the time required for each set of angles was approximately eight minutes. Without scripting, each set required approximately11 minutes. Similar time efficiency gains (approximately 25%) were found for isocenter verification measurements.

IV. DISCUSSION

This study summarizes the commissioning process of the Edge, a dedicated system for SRS/SBRT treatment. Although it offers the advanced imaging package, the 6DoF treatment couch, and intracranial radiosurgery accessory package, the beam data characteristics and mechanical parameters of the Edge are similar to the ~~TrueBeam~~TrueBeam.

Beam data from five ~~Truebeam~~TrueBeam linacs at three different institutions were previously compared [9], and we noted excellent agreement between the beam data collected on the Edge and that on the ~~Truebeam~~TrueBeam linacs. The CC04 chamber was used to scan the PDDs and profiles for the Edge while the CC 13 chamber was used for the ~~TrueBeam~~TrueBeam, and due to its smaller active volume, dose falloff in profiles for the Edge was slightly sharper than that for the ~~Truebeam~~TrueBeam. Kim et al compared PDD and cross-plane profiles of a 6MV SRS beam using four different detectors (SFD, PFD, CC01 and CC13) [35]. They showed that PDDs from all detectors were in good agreement for field sizes ranging from 1 × 1 to 6 × 6 cm^2. Diodes overestimated the dose for field sizes larger than 6 × 6 cm^2 due to lower energy, scattered photons. For profile scans, CC13 ion chamber showed a larger blurring of penumbra even for field size of 10 × 10 cm^2. A small sensitive volume detector is recommended to achieve a sharper penumbra. However,

CC01 (steel electrode) or diode are likely to measure higher dose in the tails due to the over-response to low energy, scattered photons.

The dose per pulse at the central axis is higher than off-axis due to the absence of the flattening filter. Since ion collection efficiency is a function of the dose per pulse, P_{ion} was measured and compared between the central axis and off axis for two different field sizes (1.007, 1.009, 1.010 at central axis, 2.4 cm off axis and 5.6 cm off axis respectively for 6XFFF and 1.011, 1.010, 1.009 for 10XFFF). The consistency of P_{ion} at different locations ensures there is no additional correction needed for the profile measurement.

Several challenges in small field dosimetry exist, including lack of charged particle equilibrium (CPE), overestimation of field size, perturbation of the particle fluence in the chamber and volume averaging effect of the detector etc[36]. Therefore, it is crucial to choose the correct detector considering the size, energy dependence, and perturbation etc. A new formalism has been developed ~~to~~ for the dosimetry of small field [37]. For the Edge commissioning, the machine-specific reference field is defined at 3 × 3 cm^2 since the conventional 10 × 10 cm^2 cannot be established for all detectors considering the energy dependence of the diodes and volume averaging effect of the ion chambers. The field factor $\Omega_{Q_{clin},Q_{msr}}^{f_{clin},f_{msr}}$ under the notion proposed by Alfonso et al[37], which converts the absorbed dose to water for the machine-specific reference field (3 × 3 cm^2) to the absorbed dose to water for the small clinical field, should be carefully evaluated to account for the difference of the detector response and beam quality at two different field sizes. A Monte Carlo calculated factor $k_{Q_{clin},Q_{msr}}^{f_{clin},f_{msr}}$ was recommended to correct the field factor. Several studies have been published since then to generate correction factors for various detectors from different treatment platforms[38, 39]. The diodes were shown to have an over response at small fields. A correction factor should be applied to the SFD for field sizes less than 1 × 1 cm^2 ~~for SFD~~ and the Edge detector for field sizes within 1.5 × 1.5 cm^2 [39]. This factor might also explain the 4% difference in the output factor measurements between the Edge detector and SFD for conical cones. A Monte Carlo simulation for the FFF beams may be beneficial in verifying the correction factors for stereotactic diodes at very small field sizes (< 2 cm).

There are various methods to measure the DLG: (1) measuring the distance between the radiation and geometrical field edge of a MLC defined field size, (2) matching the gap width profiles with the measured values, (3) optimizing the parameters based on treatment delivery, and (4) sweeping MLC leaves with a variety of sliding MLC gap widths[40, 41]. For the Eclipse TPS, only one DLG value can be commissioned for all different field sizes and delivery techniques. Therefore, there is a tradeoff in the optimal DLG between IMRT and RapidArc measured fields as well as the fields with different sizes and modulation. The difference

between the measured and optimized DLG values is caused by different contributions to the dose from the beam penumbra, which is a consequence of different patterns of leaf movement. Szpala et al. found out that the DLG values are a function of the distance (in the BEV) between the dose point and the leaf ending, and the width of the MLC slit[42]. Therefore calculation using a single DLG value may overestimate the measurement in the proximal penumbra, while it may underestimate the dose in the distal penumbra for RapidArc delivery[42]. For IMRT delivery, the DLG values for smaller and larger regions average out and a single value can serve as the optimal value for different widths of the MLC slits[42]. Therefore the DLG values were optimized for RapidArc delivery by evaluating the measured and calculated dose for selected spine radiosurgery cases due to the requirement of an extremely steep dose gradient. The adjustments did not have much impact on the IMRT delivery. The dose calculation accuracy was further validated in a more comprehensive manner using test cases representative of various clinical treatment sites.

Tissue maximum ratios (TMR) and off axis rations ratios(OAR) are used for the cone-based dose calculation. TMR values can be measured by draining or filling water in a 3D water tank or derived from PDD curves. It is challenging to use the conventional conversion methods since phantom scatter factors for small fields are difficult to measure. Battum et al. proposed to obtain TMR values from PDD curves and total scatter factors _ENREF_43[43]. A depth dose curve corrected for source detector distance was generated from existing PDD curves and the dose at each depth and field size was fitted by a double exponential function. TMR was then calculated by taking the ratio of the dose at the depth of interest and the reference depth. They reported the agreement between calculated and measured TMR was within 2%. TMR values were spot checked on the Edge system at nine points for each cone and compared against the converted data. The difference was within 2% except at 20 cm, the deepest depth. Larger discrepancies were noted at depths beyond 20 cm, which is generally greater than the maximum depth required for intracranial SRS treatment. This method can be considered an alternative option to obtain TMR values for cones when a precise TMR measurement is not available from the water tank.

Conical cones may provide a sharper beam penumbra than the MLCs since the cone is closer to the isocenter and more transmission occurs at the round leaf ends of the MLCs. The beam penumbra for the cones is a function of depth, cone size, energy. It increases as the cone size, depth or energy increases. The beam penumbra increases faster for the 90-10% value than for the 80-20% value as shown in- Figure 8.

The 6DoF (PerfectPitch™) couch top is equipped with rails, which will lead to errors in the delivered dose, if the rails are not properly accounted for the in treatment plan. This is especially important in the context of

spine SRS, where highly modulated, posterior beams are used and the isodose fall off from 90% to 50% line is on the order of 3 mm. Therefore, the attenuation effect of the rails and couch tops should be measured. A proper couch model should be established in the treatment planning system according to recommendations from AAPM Task Group report No. 176 [44]. By taking CT scans of the couch top prior to installation on the treatment unit, couch models can be developed along with a setup for future planning and delivery to a QA phantom. In this study, such a couch model was incorporated for all the test plans related to the Edge commissioning, phantom QA and patient planning. The couch model is also used for routine patient treatment planning.

To optimize use of the couch model for RapidArc delivery, one solution is to place both couch rails in the ‘in’ position and start the arc at oblique angles to avoid the beam traversing through the rails. However, the rigidity of the couch insert should be carefully evaluated in the lateral direction (patient left and right) for such a configuration. The deviation was linear with lateral translation, due mainly to the rigidity of the couch moving mechanism.

Since target localization may incorporate single or multiple imaging modalities and 6DoF couch correction, end-to-end tests were designed to evaluate the coincidence of each imaging modality with the radiation isocenter, the accuracy of 3D-3D and 2D-3D image registration, the precision of 6DoF correction, and the coincidence of gantry, collimator and couch axes with the radiation isocenter. The laser and crosshair alignment should also be checked after the phantom localization. By performing the Winston-Lutz test on a daily basis, the localization accuracy can be accessed and deviations can be easily identified to trigger further action, including imaging system calibration, couch precision test or LINAC mechanical check etc.

V. CONCLUSION

We present technical aspects related to comprehensive commissioning and assessment of localization and delivery accuracy of a novel, LINAC-based SRS/SBRT-based treatment system (The Edge, Varian Medical Systems, Palo Alto, CA). We have demonstrated that the beam characteristics and localization accuracy of this system are well suited for the frameless, LINAC-based SRS, SBRT treatments, and other general treatment indications in radiation oncology.

VI. ACKNOWLEDGEMENTS

This work has been supported in part by a research grant from Varian Medical Systems, Palo Alto, CA.

REFERENCES

[1] L. Leksell, "Sterotaxic radiosurgery in trigeminal neuralgia," Acta chirurgica Scandinavica **137**, 311-314 (1971).

[2] J.R. Adler, Jr., S.D. Chang, M.J. Murphy, J. Doty, P. Geis, S.L. Hancock, "The Cyberknife: a frameless robotic system for radiosurgery," Stereotactic and functional neurosurgery **69**, 124-128 (1997).

[3] S. Ryu, F. Fang Yin, J. Rock, J. Zhu, A. Chu, E. Kagan, L. Rogers, M. Ajlouni, M. Rosenblum, J.H. Kim, "Image-guided and intensity-modulated radiosurgery for patients with spinal metastasis," Cancer **97**, 2013-2018 (2003).

[4] M. Garcia-Barros, F. Paris, C. Cordon-Cardo, D. Lyden, S. Rafii, A. Haimovitz-Friedman, Z. Fuks, R. Kolesnick, "Tumor response to radiotherapy regulated by endothelial cell apoptosis," Science **300**, 1155-1159 (2003).

[5] O.O. Betti, Y.E. Derechinsky, "Irradiations stereotaxiques multifaisceaux," Neurochirurgie **28**, 55-56 (1982).

[6] W. Lutz, K.R. Winston, N. Maleki, "A system for stereotactic radiosurgery with a linear accelerator," Int J Radiat Oncol Biol Phys **14**, 373-381 (1988).

[7] W.M. Saunders, K.R. Winston, R.L. Siddon, G.H. Svensson, P.K. Kijewski, R.K. Rice, J.L. Hansen, N.H. Barth, "Radiosurgery for arteriovenous malformations of the brain using a standard linear accelerator: rationale and technique," Int J Radiat Oncol Biol Phys **15**, 441-447 (1988).

[8] I.J. Das, M.B. Downes, B.W. Corn, W.J. Curran, M. Werner-Wasik, D.W. Andrews, "Characteristics of a dedicated linear accelerator-based stereotactic radiosurgery-radiotherapy unit," Radiother Oncol **38**, 61-68 (1996).

[9] C. Glide-Hurst, M. Bellon, R. Foster, C. Altunbas, M. Speiser, M. Altman, D. Westerly, N. Wen, B. Zhao, M. Miften, I.J. Chetty, T. Solberg, "Commissioning of the Varian TrueBeam linear accelerator: A multi-institutional study," Med Phys **40**, 031719 (2013).

[10] F.F. Yin, J. Zhu, H. Yan, H. Gaun, R. Hammoud, S. Ryu, J.H. Kim, "Dosimetric characteristics of Novalis shaped beam surgery unit," Med Phys **29**, 1729-1738 (2002).

[11] D. Georg, T. Knoos, B. McClean, "Current status and future perspective of flattening filter free photon beams," Med Phys **38**, 1280-1293 (2011).

[12] Z. Chang, Z. Wang, Q.J. Wu, H. Yan, J. Bowsher, J. Zhang, F.F. Yin, "Dosimetric characteristics of novalis Tx system with high definition multileaf collimator," Med Phys **35**, 4460-4463 (2008).

[13] T.D. Solberg, K.L. Boedeker, R. Fogg, M.T. Selch, A.A. DeSalles, "Dynamic arc radiosurgery field shaping: a comparison with static field conformal and noncoplanar circular arcs," Int J Radiat Oncol Biol Phys **49**, 1481-1491 (2001).

[14] J.Y. Jin, Q. Chen, R. Jin, J. Rock, J. Anderson, S. Li, B. Movsas, S. Ryu, "Technical and clinical experience with spine radiosurgery: a new technology for management of localized spine metastases," Technology in cancer research & treatment **6**, 127-133 (2007).

[15] C. Audet, B.A. Poffenbarger, P. Chang, P.S. Jackson, R.E. Lundahl, S.I. Ryu, G.R. Ray, "Evaluation of volumetric modulated arc therapy for cranial radiosurgery using multiple noncoplanar arcs," Med Phys **38**, 5863-5872 (2011).

[16] Y. Huang, K. Chin, J.R. Robbins, J. Kim, H. Li, H. Amro, I.J. Chetty, J. Gordon, S. Ryu, "Radiosurgery of multiple brain metastases with single-isocenter dynamic conformal arcs (SIDCA)," Radiother Oncol2014).

17 L.S. Johnson, B.D. Milliken, S.W. Hadley, C.A. Pelizzari, D.J. Haraf, G.T. Chen, "Initial clinical experience with a video-based patient positioning system," Int J Radiat Oncol Biol Phys **45**, 205-213 (1999).

18 A.S. Shiu, E.L. Chang, J.S. Ye, M. Lii, L.D. Rhines, E. Mendel, J. Weinberg, S. Singh, M.H. Maor, R. Mohan, J.D. Cox, "Near simultaneous computed tomography image-guided stereotactic spinal radiotherapy: an emerging paradigm for achieving true stereotaxy," Int J Radiat Oncol Biol Phys **57**, 605-613 (2003).

19 T.C. Ryken, S.L. Meeks, V. Traynelis, J. Haller, L.G. Bouchet, F.J. Bova, E.C. Pennington, J.M. Buatti, "Ultrasonographic guidance for spinal extracranial radiosurgery: technique and application for metastatic spinal lesions," Neurosurgical focus **11**, e8 (2001).

20 J. Kim, J.Y. Jin, N. Walls, T. Nurushev, B. Movsas, I.J. Chetty, S. Ryu, "Image-guided localization accuracy of stereoscopic planar and volumetric imaging methods for stereotactic radiation surgery and stereotactic body radiation therapy: a phantom study," Int J Radiat Oncol Biol Phys **79**, 1588-1596 (2011).

21 H. Yan, F.F. Yin, J.H. Kim, "A phantom study on the positioning accuracy of the Novalis Body system," Med Phys **30**, 3052-3060 (2003).

22 R. Nath, P.J. Biggs, F.J. Bova, C.C. Ling, J.A. Purdy, J. van de Geijn, M.S. Weinhous, "AAPM code of practice for radiotherapy accelerators: report of AAPM Radiation Therapy Task Group No. 45," Med Phys **21**, 1093-1121 (1994).

23 I.J. Das, C.W. Cheng, R.J. Watts, A. Ahnesjo, J. Gibbons, X.A. Li, J. Lowenstein, R.K. Mitra, W.E. Simon, T.C. Zhu, ~~T.G.o.t.T.P.C.o.t. AAPM,~~ "Accelerator beam data commissioning equipment and procedures: report of the TG-106 of the Therapy Physics Committee of the AAPM," Med Phys **35**, 4186-4215 (2008).

24 V.P. T. Torsti; L. Korhonen, "Using Varian photon beam source model for dose calculation of small fields Clinical Prospectives," 2013).

25 T. LoSasso, C.S. Chui, C.C. Ling, "Physical and dosimetric aspects of a multileaf collimation system used in the dynamic mode for implementing intensity modulated radiotherapy," Med Phys **25**, 1919-1927 (1998).

26 F. Khan, *The Physics of Radiation Therapy*. (Williams & Wilkins, 1984).

27 D. Schmidhalter, M.K. Fix, M. Wyss, N. Schaer, P. Munro, S. Scheib, P. Kunz, P. Manser, "Evaluation of a new six degrees of freedom couch for radiation therapy," Med Phys **40**, 111710 (2013).

28 G.A. Ezzell, J.W. Burmeister, N. Dogan, T.J. LoSasso, J.G. Mechalakos, D. Mihailidis, A. Molineu, J.R. Palta, C.R. Ramsey, B.J. Salter, J. Shi, P. Xia, N.J. Yue, Y. Xiao, "IMRT commissioning: multiple institution planning and dosimetry comparisons, a report from AAPM Task Group 119," Med Phys **36**, 5359-5373 (2009).

29 G.M. Clark, R.A. Popple, B.M. Prendergast, S.A. Spencer, E.M. Thomas, J.G. Stewart, B.L. Guthrie, J.M. Markert, J.B. Fiveash, "Plan quality and treatment planning technique for single isocenter cranial radiosurgery with volumetric modulated arc therapy," Practical radiation oncology **2**, 306-313 (2012).

30 N. Wen, B. Zhao, J. Kim, K. Chin-Snyder, M. Bellon, C. Glide-Hurst, K. Barton, D. Chen, I.J. Chetty, "IMRT and RapidArc commissioning of a TrueBeam linear accelerator using TG-119 protocol cases," J Appl Clin Med Phys **15**, 4843 (2014).

31 E.E. Klein, J. Hanley, J. Bayouth, F.F. Yin, W. Simon, S. Dresser, C. Serago, F. Aguirre, L. Ma, B. Arjomandy, C. Liu, C. Sandin, T. Holmes, A.A.o.P.i.M. Task Group, "Task Group 142 report: quality assurance of medical accelerators," Med Phys **36**, 4197-4212 (2009).

[32] F. Ponisch, U. Titt, O.N. Vassiliev, S.F. Kry, R. Mohan, "Properties of unflattened photon beams shaped by a multileaf collimator," Med Phys **33**, 1738-1746 (2006).

[33] S. Dieterich, G.W. Sherouse, "Experimental comparison of seven commercial dosimetry diodes for measurement of stereotactic radiosurgery cone factors," Med Phys **38**, 4166-4173 (2011).

[34] T.D. Solberg, J.M. Balter, S.H. Benedict, B.A. Fraass, B. Kavanagh, C. Miyamoto, T. Pawlicki, L. Potters, Y. Yamada, "Quality and safety considerations in stereotactic radiosurgery and stereotactic body radiation therapy: Executive summary," Practical radiation oncology **2**, 2-9 (2012).

[35] J. Kim, N. Wen, J.Y. Jin, N. Walls, S. Kim, H. Li, L. Ren, Y. Huang, A. Doemer, K. Faber, T. Kunkel, A. Balawi, K. Garbarino, K. Levin, S. Patel, M. Ajlouni, B. Miller, T. Nurushev, C. Huntzinger, R. Schulz, I.J. Chetty, B. Movsas, S. Ryu, "Clinical commissioning and use of the Novalis Tx linear accelerator for SRS and SBRT," J Appl Clin Med Phys **13**, 3729 (2012).

[36] I.J. Das, G.X. Ding, A. Ahnesjo, "Small fields: nonequilibrium radiation dosimetry," Med Phys **35**, 206-215 (2008).

[37] R. Alfonso, P. Andreo, R. Capote, M.S. Huq, W. Kilby, P. Kjall, T.R. Mackie, H. Palmans, K. Rosser, J. Seuntjens, W. Ullrich, S. Vatnitsky, "A new formalism for reference dosimetry of small and nonstandard fields," Med Phys **35**, 5179-5186 (2008).

[38] G. Cranmer-Sargison, S. Weston, J.A. Evans, N.P. Sidhu, D.I. Thwaites, "Implementing a newly proposed Monte Carlo based small field dosimetry formalism for a comprehensive set of diode detectors," Med Phys **38**, 6592-6602 (2011).

[39] P. Francescon, S. Cora, N. Satariano, "Calculation of k(Q(clin),Q(msr)) (f(clin),f(msr)) for several small detectors and for two linear accelerators using Monte Carlo simulations," Med Phys **38**, 6513-6527 (2011).

[40] I. Chauvet, A. Petitfils, C. Lehobey, J.Y. Kristner, Y. Brunet, R. Lembrez, G. Gaboriaud, A. Mazal, S. Zefkili, J.C. Rosenwald, "The sliding slit test for dynamic IMRT: a useful tool for adjustment of MLC related parameters," Phys Med Biol **50**, 563-580 (2005).

[41] K.N. Kielar, E. Mok, A. Hsu, L. Wang, G. Luxton, "Verification of dosimetric accuracy on the TrueBeam STx: rounded leaf effect of the high definition MLC," Med Phys **39**, 6360-6371 (2012).

[42] S. Szpala, F. Cao, K. Kohli, "On using the dosimetric leaf gap to model the rounded leaf ends in VMAT/RapidArc plans," J Appl Clin Med Phys **15**, 4484 (2014).

[43] L.J. van Battum, M. Essers, P.R. Storchi, "Conversion of measured percentage depth dose to tissue maximum ratio values in stereotactic radiotherapy," Phys Med Biol **47**, 3289-3300 (2002).

[44] A.J. Olch, L. Gerig, H. Li, I. Mihaylov, A. Morgan, "Dosimetric effects caused by couch tops and immobilization devices: report of AAPM Task Group 176," Med Phys **41**, 061501 (2014).

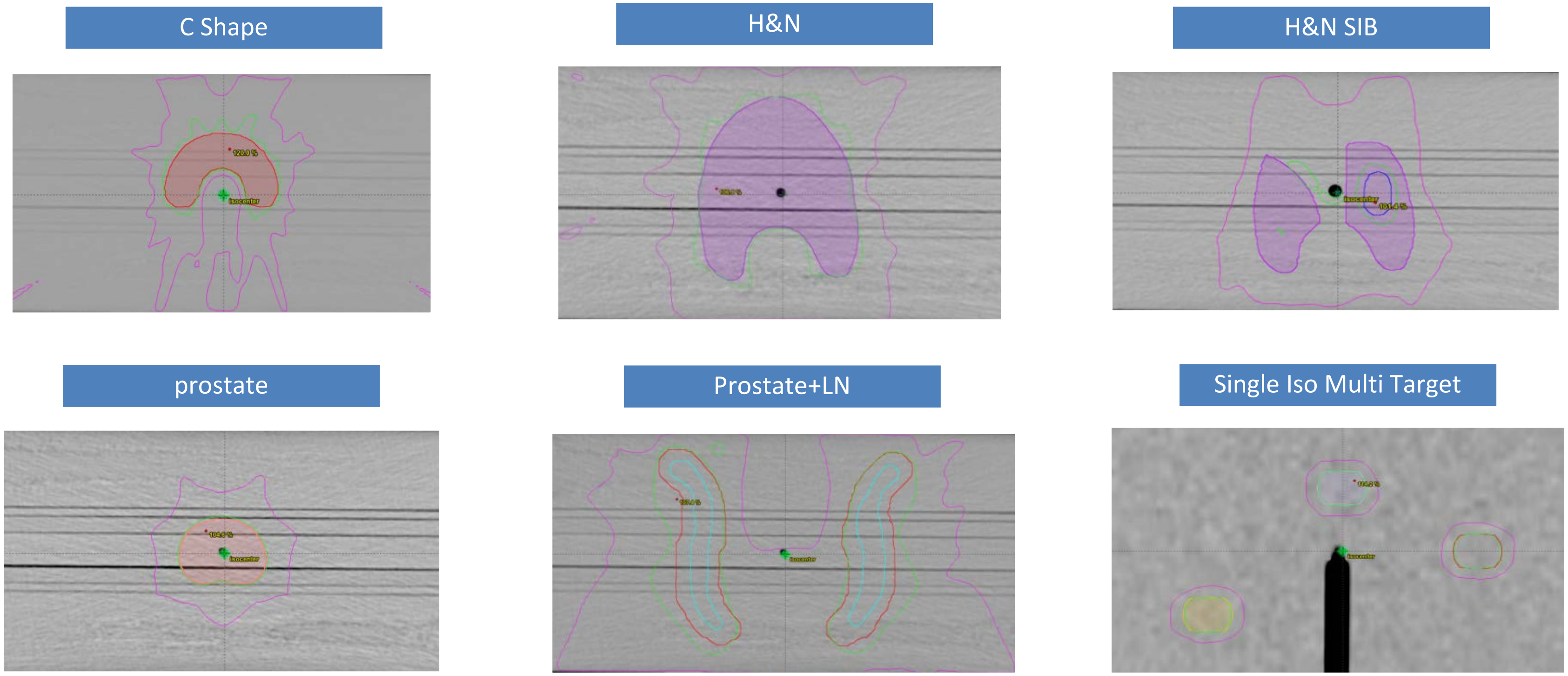

Figure 1. C shape plan: C shape target planned with IMRT using 6X-FFF; H&N plan: HN PTV target with the cord and parotid glands planned with IMRT using 6XFFF; H&N SIB plan: HN PTV50 (shaded magenta) and PTV60 (blue) targets with the cord and parotid glands planned with IMRT using 6XFFF; Prostate plan: prostate PTV (pink) planned with rectum and bladder with IMRT using 6X-FFF; Prostate+LN plan: prostate+LN(blue) PTV target (red) with rectum and bladder planned with IMRT using 6X-FFF; Single Iso Multi Target plan: 3 targets (orange, purple, and red) planned with IMRT using 6X-FFF. The isodose lines represent 95% (green) and 50% (magenta) prescription dose.

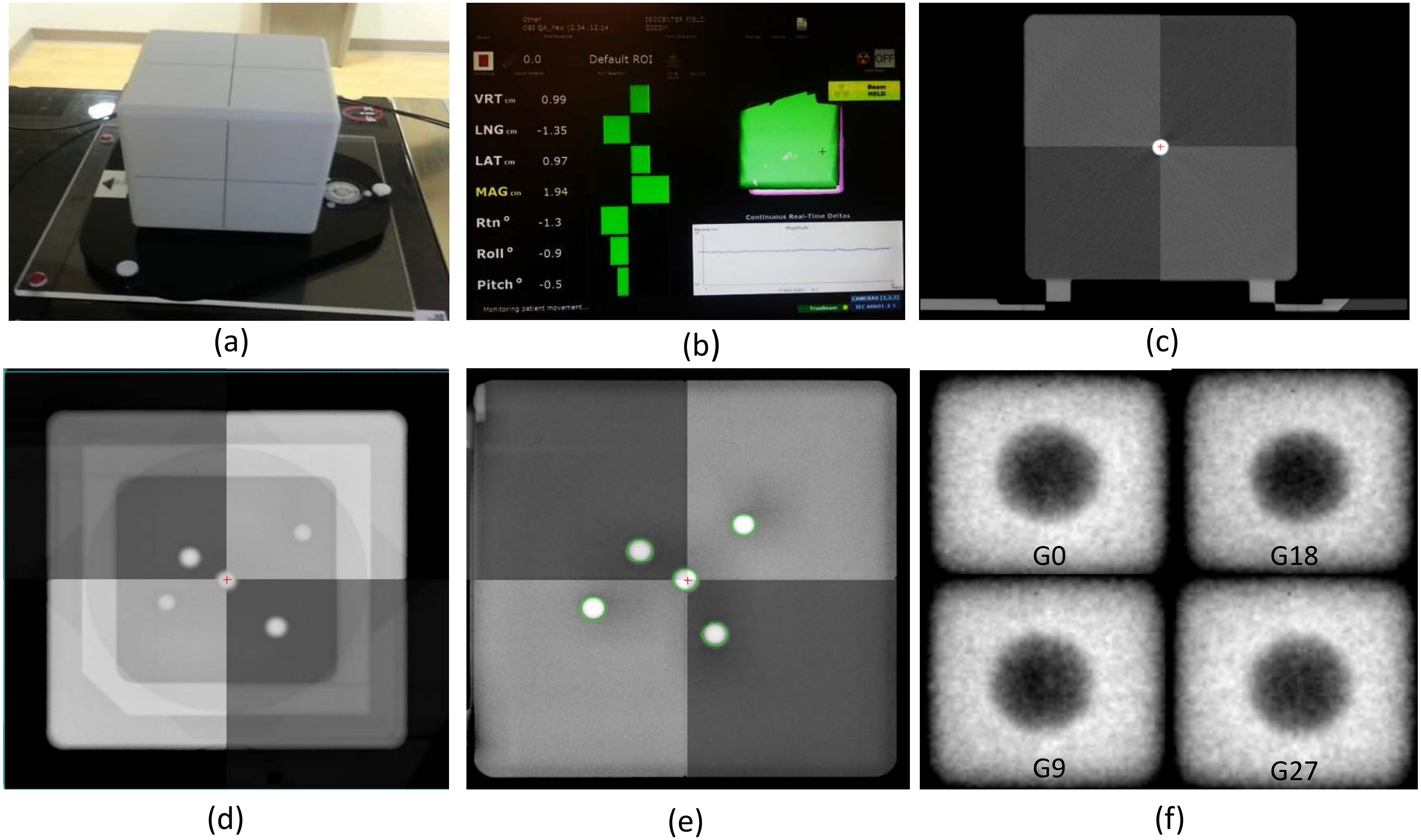

Figure 2. (a) The OSMS QA phantom sitting on top of an acrylic base plate. (b) The localization of phantom surface using the OSMS system. The difference (delta) between the current position of the OSMS phantom and its reference position is shown in 6DoF. (c) The six degree automatic fusion between planning CT and CBCT after adjusting the contrast of the acquired image and reference image to achieve optimal visualization of the BBs. An orthogonal MV (d)/KV (e) image set is taken and 2D-3D image fusion is performed to quantify the residual error. (f) Four representative MLC defined portal images of the Winston-Lutz test.

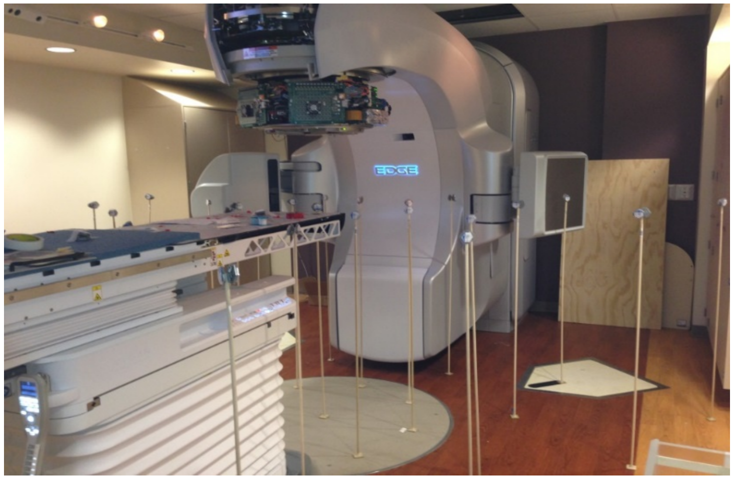

(a)

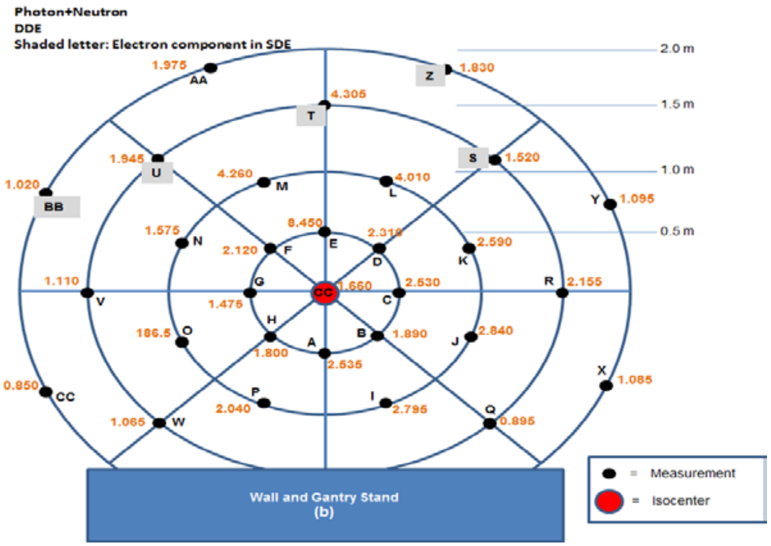

Photon+Neutron
DDE
Shaded letter: Electron component in SDE
2.0 m
1.5 m
1.0 m
0.5 m
1.975 AA
Z 1.830
4.305 T
1.945 U
S 1.520
1.020 BB
Y 1.095
4.260 M
4.010 L
1.575 N
8.450
2.120 F
E
2.310 D
K 2.590
1.110 V
G
CC 1.660
2.530 C
R 2.155
1.475
H
186.5 O
A
B 1.890
J 2.840
1.800
2.535
0.850 CC
X 1.085
P 2.040
I 2.795
Q 0.895
1.065 W
Wall and Gantry Stand
(b)
= Measurement
= Isocenter

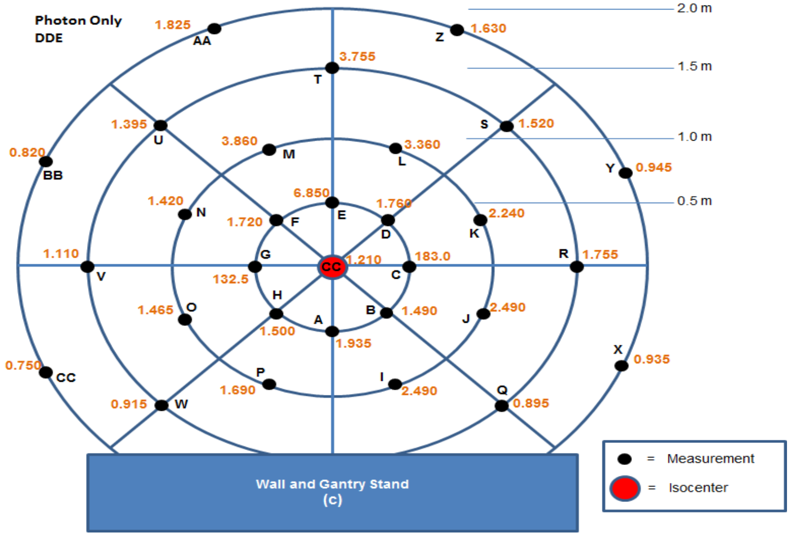

Photon Only
DDE
2.0 m
1.5 m
1.0 m
0.5 m
1.825 AA
Z 1.630
3.755 T
1.395 U
S 1.520
0.820 BB
3.860 M
3.360 L
Y 0.945
1.420 N
6.850 E
1.760 D
1.720 F
2.240 K
1.110 V
G
CC 1.210
183.0 C
R 1.755
132.5
H
1.465 O
A
B 1.490
J 2.490
1.500
1.935
0.750 CC
X 0.935
P 1.690
I 2.490
Q 0.895
0.915 W
Wall and Gantry Stand
(c)
= Measurement
= Isocenter

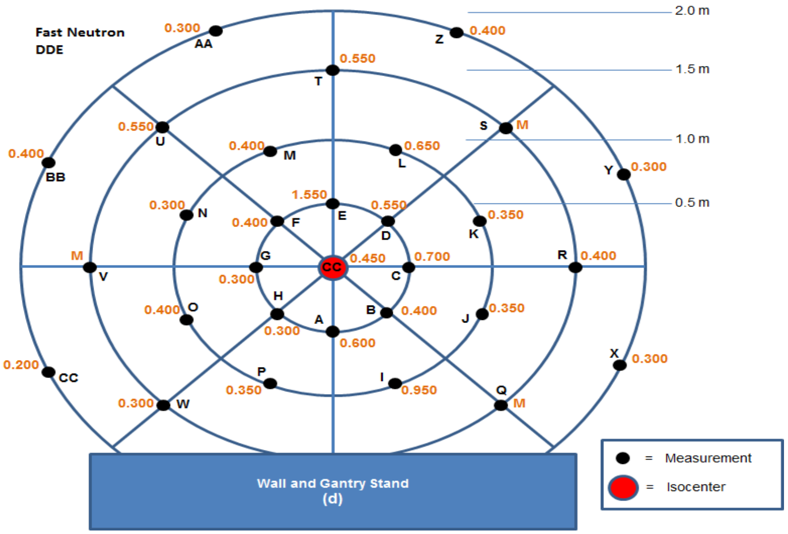

Fast Neutron
DDE
2.0 m
1.5 m
1.0 m
0.5 m
0.300 AA
Z 0.400
0.550 T
0.550 U
S M
0.400 BB
0.400 M
0.650 L
Y 0.300
0.300 N
1.550 E
0.550 D
0.400 F
K 0.350
M V
G
CC 0.450
0.700 C
R 0.400
0.300
H
0.400 O
A
B 0.400
J 0.350
0.300
0.600
0.200 CC
X 0.300
P 0.350
I 0.950
Q M
0.300 W
Wall and Gantry Stand
(d)
= Measurement
= Isocenter

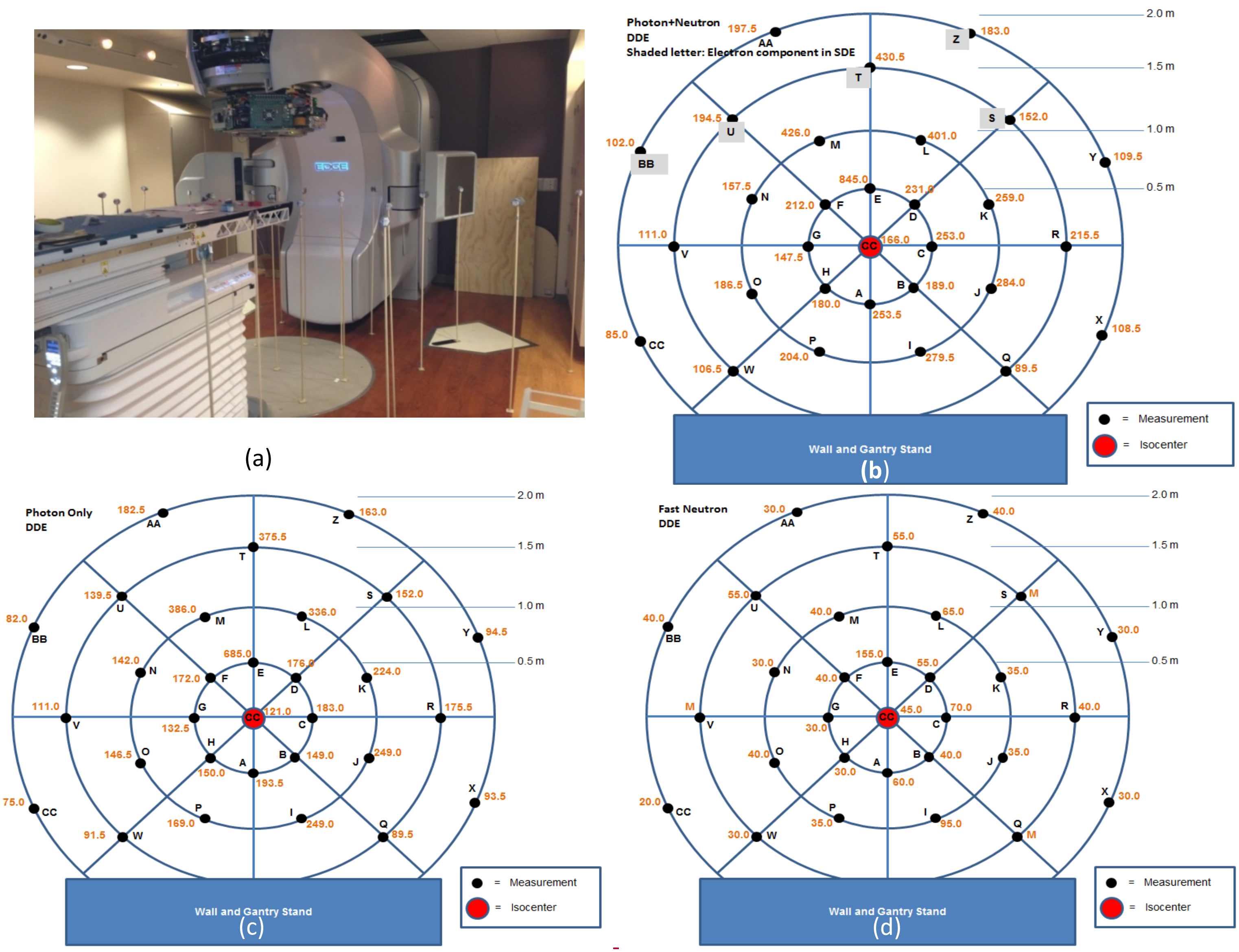

Figure 3. (a) The placement of the T series dosimeters around a 2 m radius circular plane. The deep dose equivalent map of photon and neutron combined (b), photon only (c) and fast neutron only (d). The maximum measured head leakage dose was 8.45, 6.85 and 1.55 m~~rem~~Sv respectively, all located at point E, 0.5 m toward the couch direction.

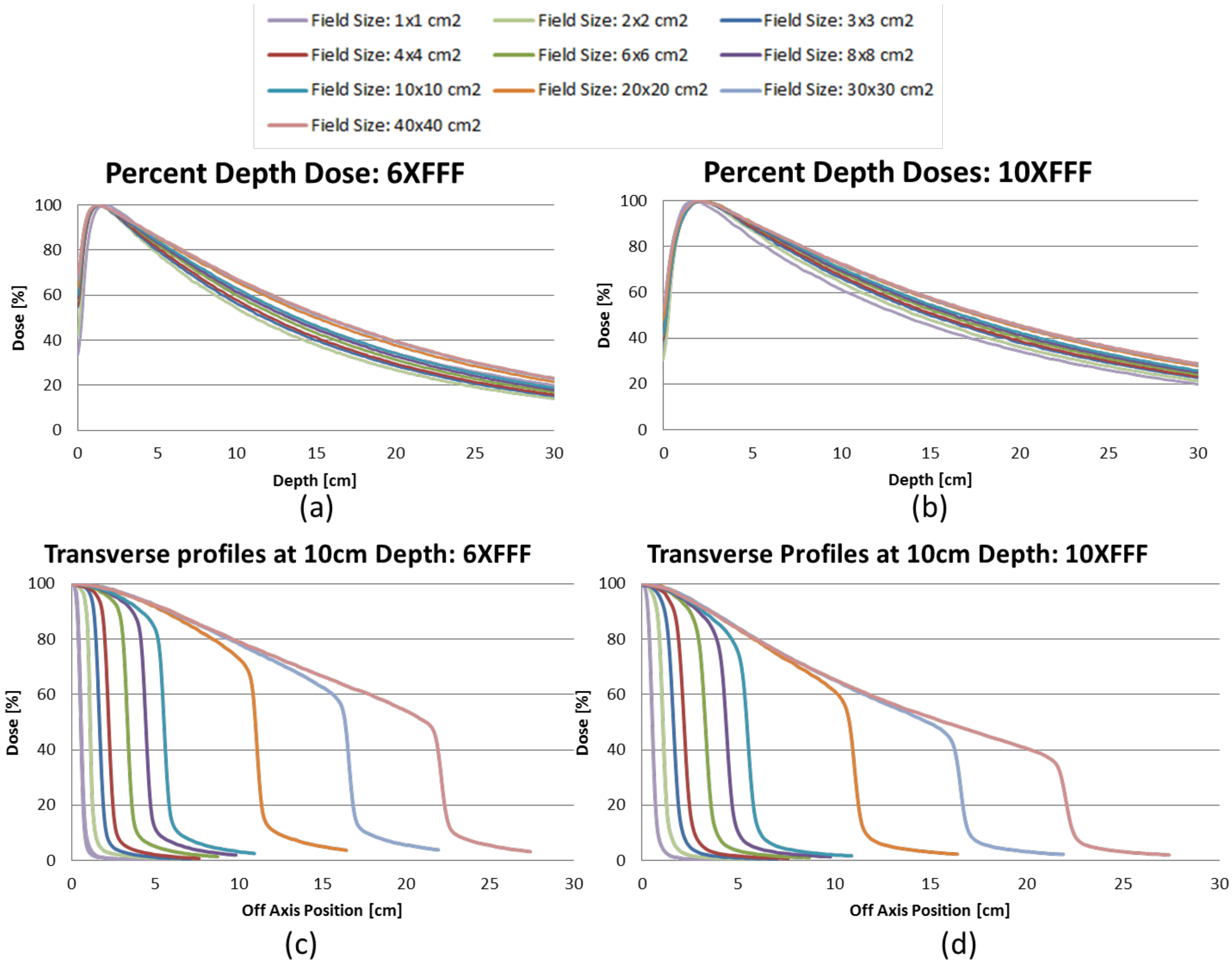

Figure 4. PDD curves normalized at Dmax for 6XFFF (a) and 10XFFF (b) for the field sizes ranging from 1 × 1 to 40 × 40 cm^2. The cross-plane profiles measured at 10 cm depth for all 10~~5~~ field sizes for 6XFFF (c) and 10XFFF (d). A CC04 cylindrical chamber was used for field sizes greater than 2 × 2 cm^2 using the 400 MU/min dose rate and the SFD was used for field sizes 1 × 1 cm^2 and 2 × 2 cm^2 using the maximum dose rate. The curves are normalized to 100% on the central axis.

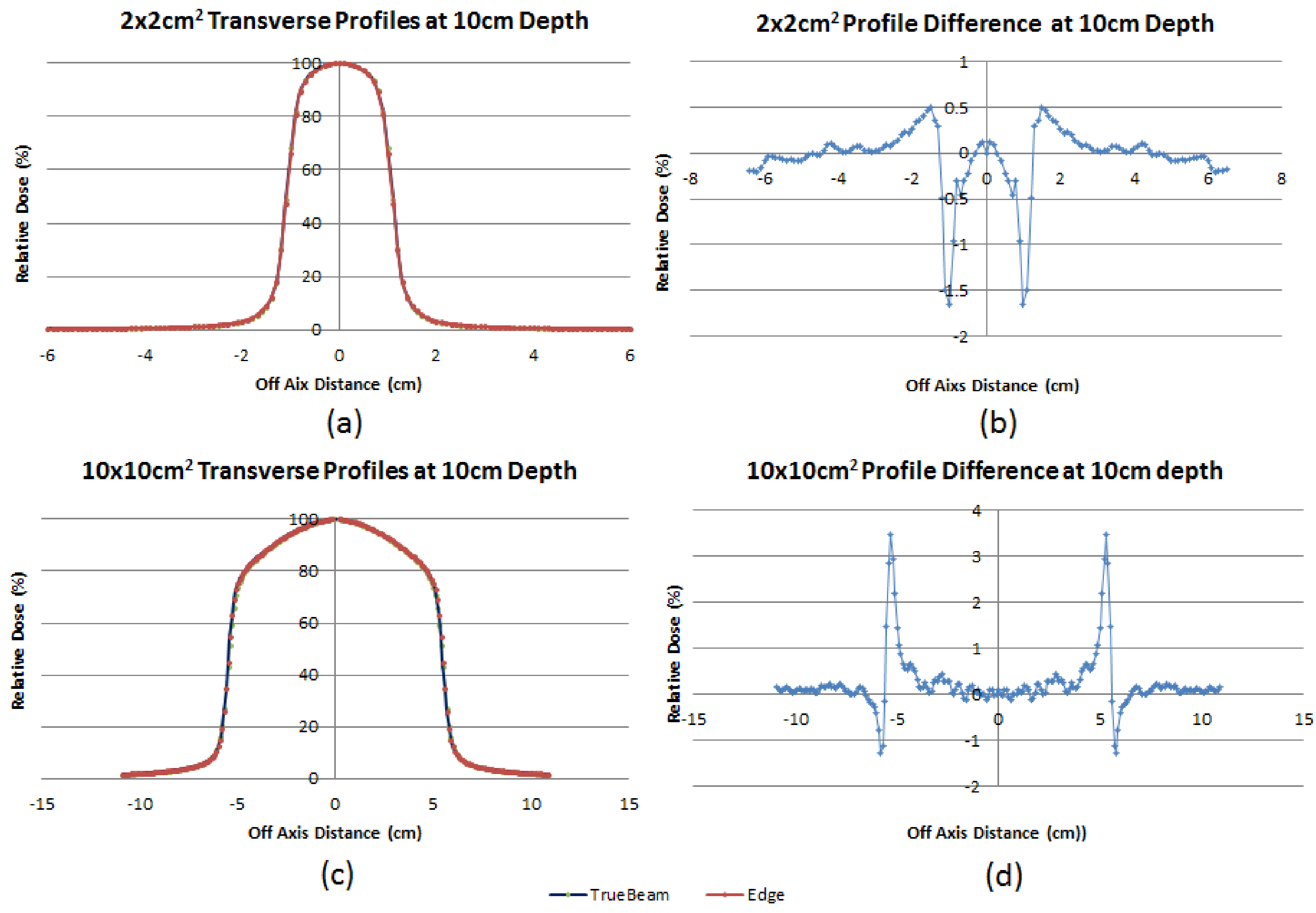

Figure 5. Comparison of profile curves between the Edge and the ~~TrueBeam~~TrueBeam for two representative fields using 10XFFF: 2 × 2 cm^2 and 10 × 10 cm^2. The profiles between the Edge and the ~~TrueBeam~~TrueBeam were practically the same with slightly sharper penumbra obtained on the Edge at all the depths.

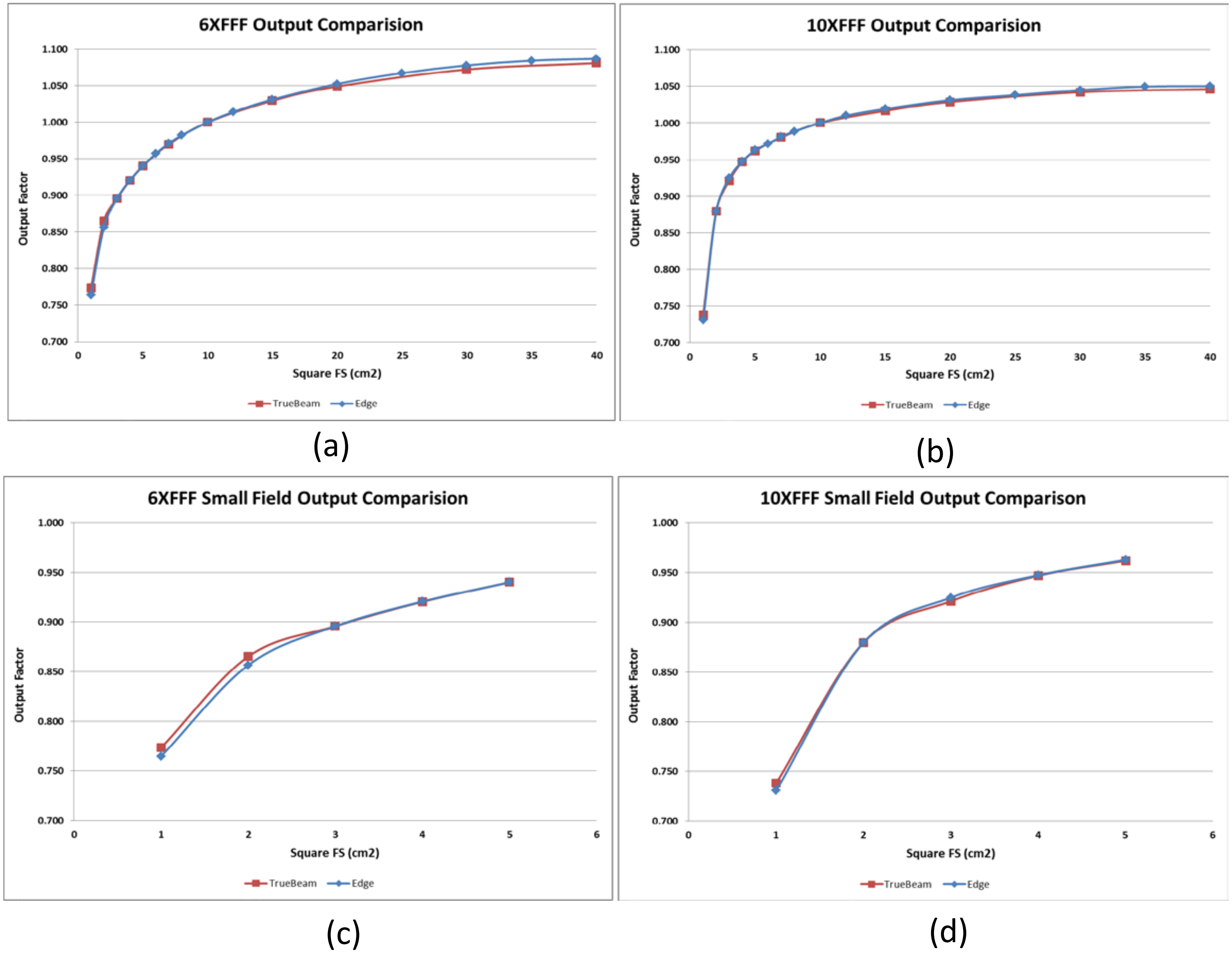

Figure 6. Comparison of Output factors between the Edge and ~~TrueBeam~~TrueBeam for symmetrical fields ranging from 1 × 1 cm^2 to 40 × 40 cm^2 for 6XFFF (a) and 10XFFF (b). The figures are magnified for small field sizes in (c) and (d).

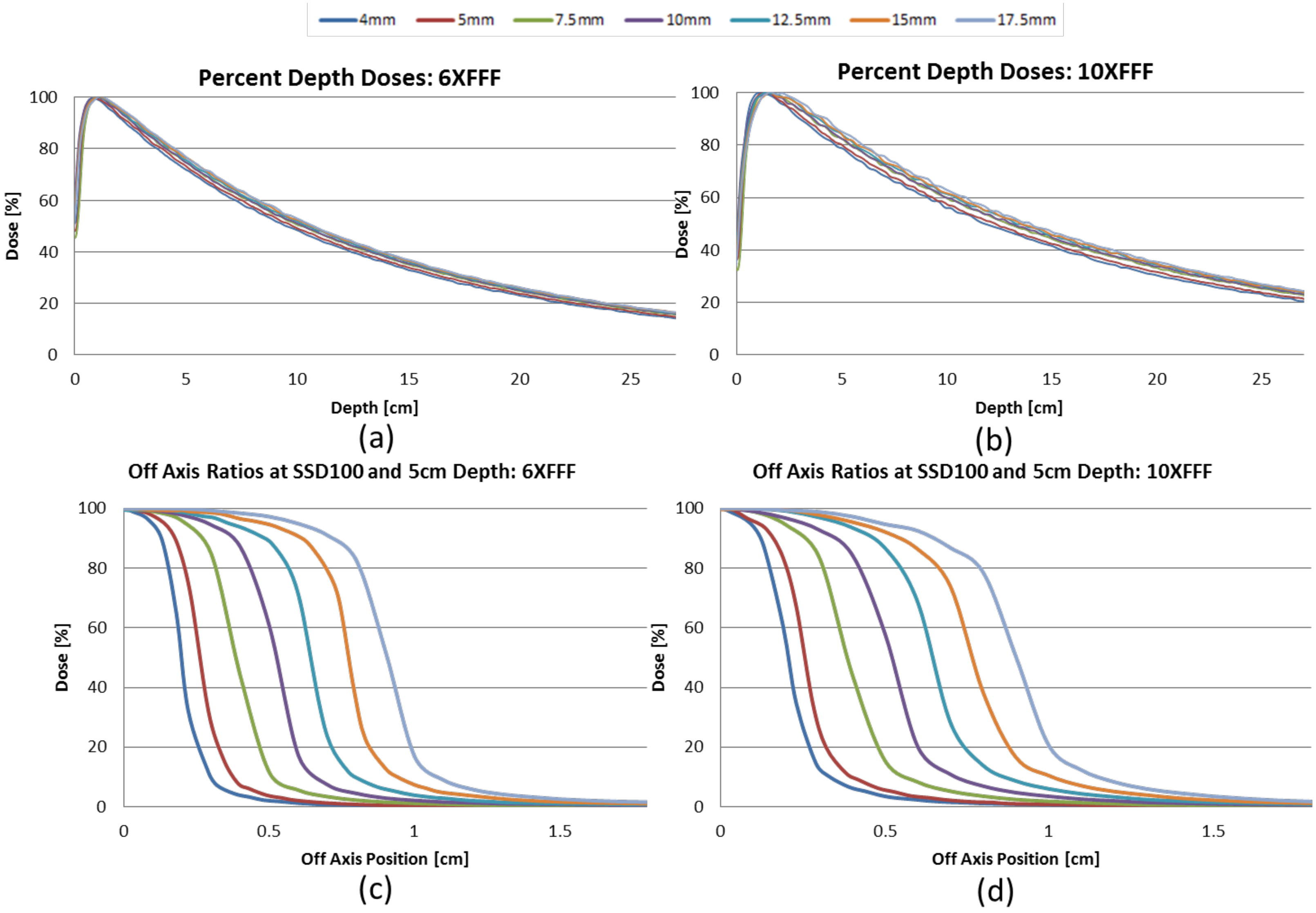

Figure 7. PDD curves normalized at Dmax for 6XFFF (a) and 10XFFF (b) for the conical cones ranging from 4 mm to 17.5mm. The off-axis ratio measured at 5 cm depth, 100 cm SSD for 6XFFF (c) and 10XFFF (d). The curves are normalized to 100% on the central axis.

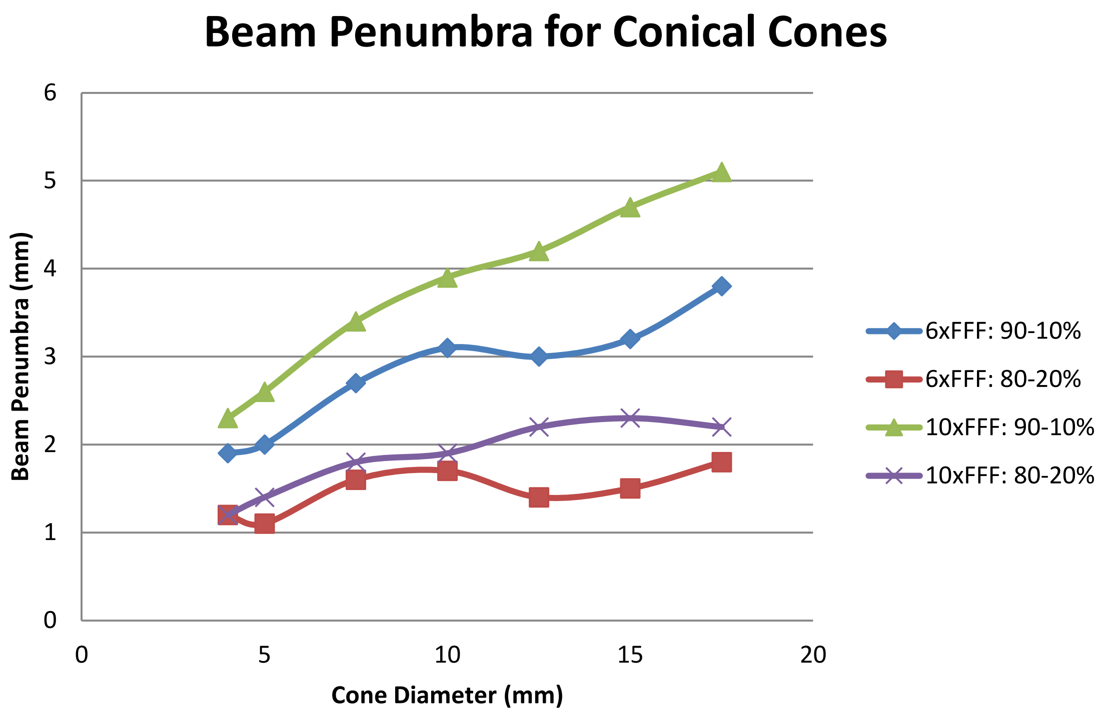

Figure 8. The beam penumbra (width between 90% - 10% and 80% - 20%) increases as the diameter of the cone increases for both energies. The beam penumbra increases faster for the 90-10% value than for the 80-20% value.

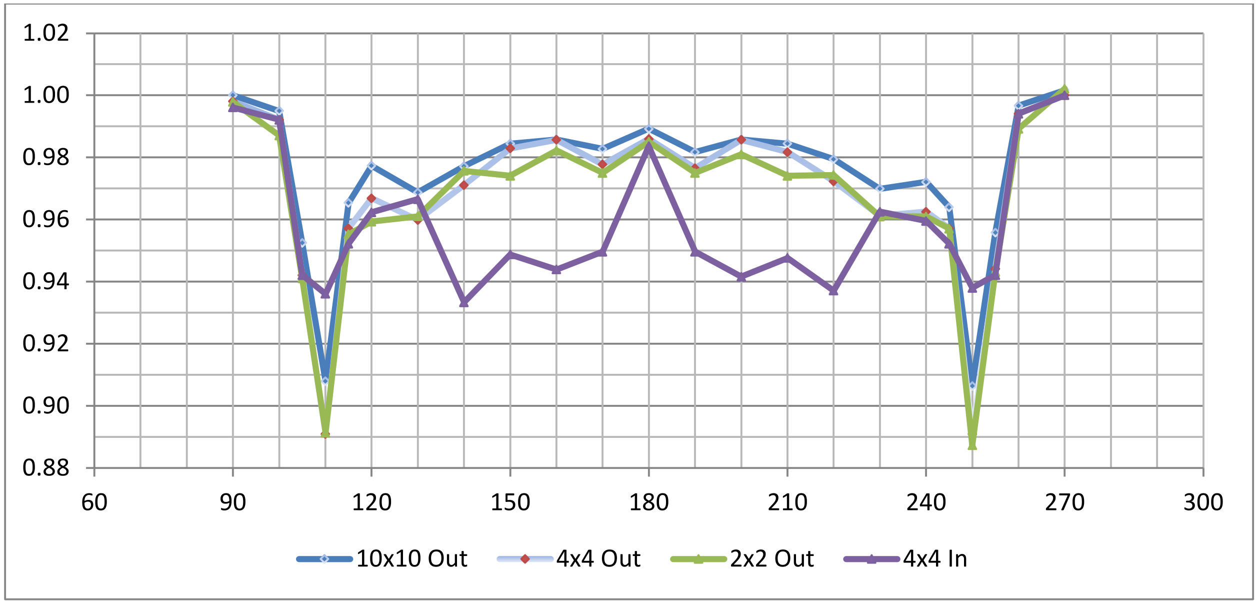

Figure 9: The relative attenuation (x axis) for the KV~~v~~ue couch at various gantry angles (y axis) ranging from 90° to 270° using 6XFFF beam at 3 different field sizes. Relative attenuation is greatest in a small window of oblique entry. The attenuation in positioning of the rails in ‘out’ and ‘in’ positions was studied using a 4 × 4 cm^2 field size.

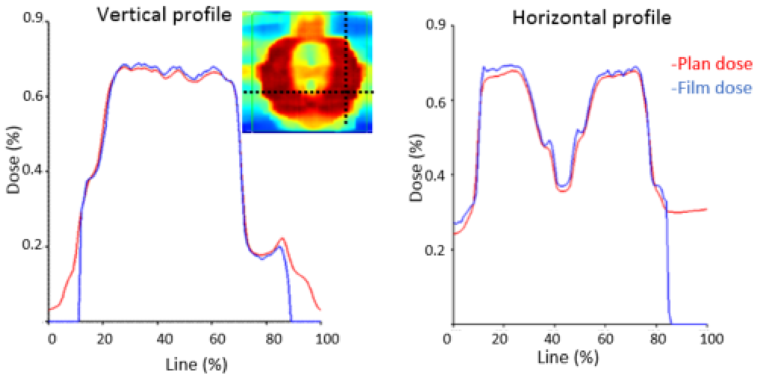

6XFFF Prostate LN IMRT (low dose)

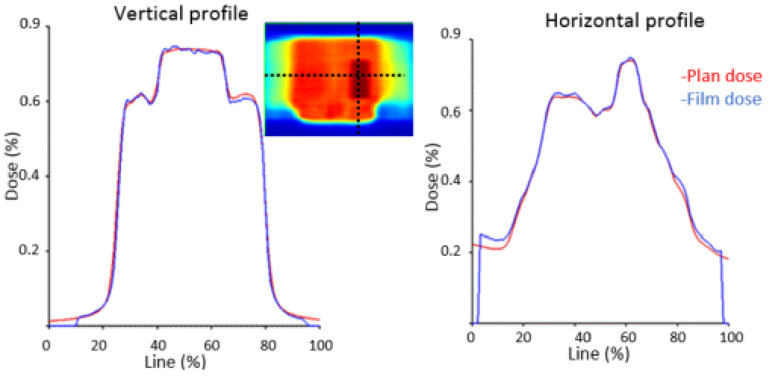

10XFFF H&NSIB IMRT (PTV)

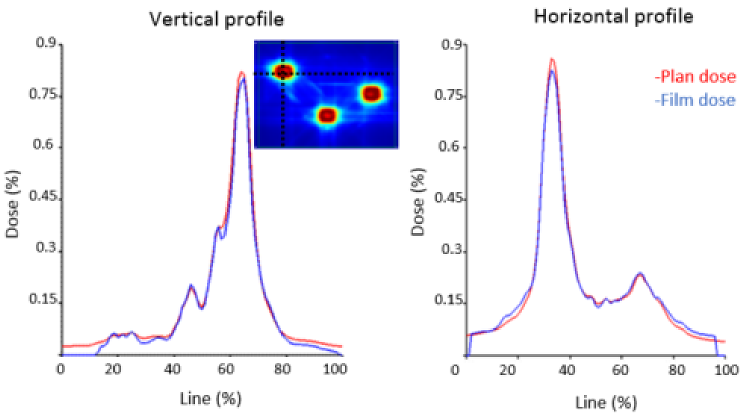

6XFFF SIMT RapidArc

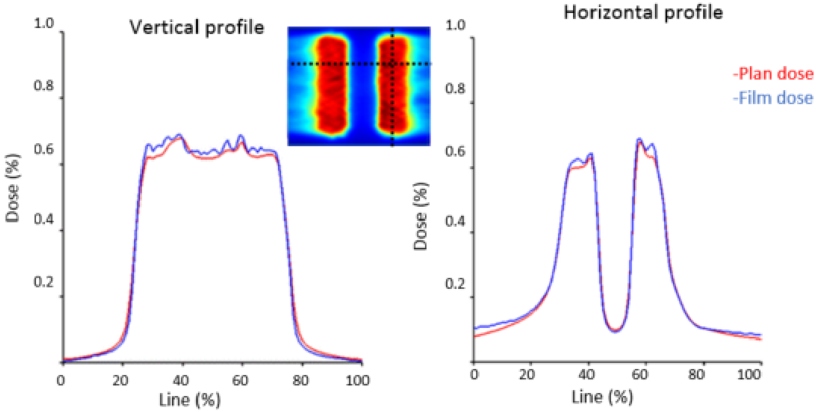

10XFFF C Shape RapidArc (low dose)

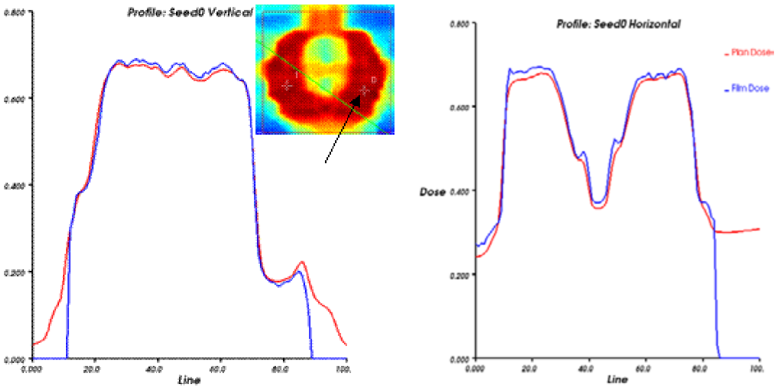

6XFFF Prostate LN IMRT (low

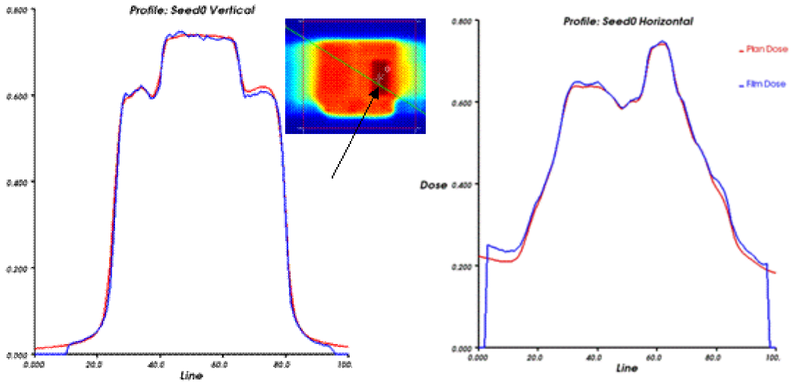

10XFFF H&NSIB IMRT (PTV)

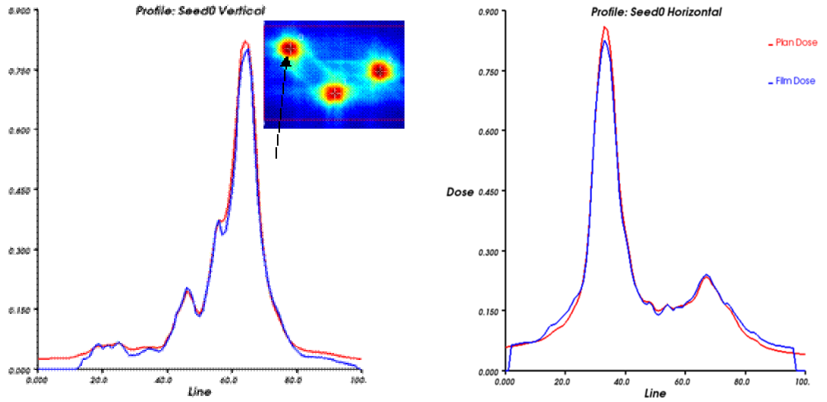

6XFFF SIMT

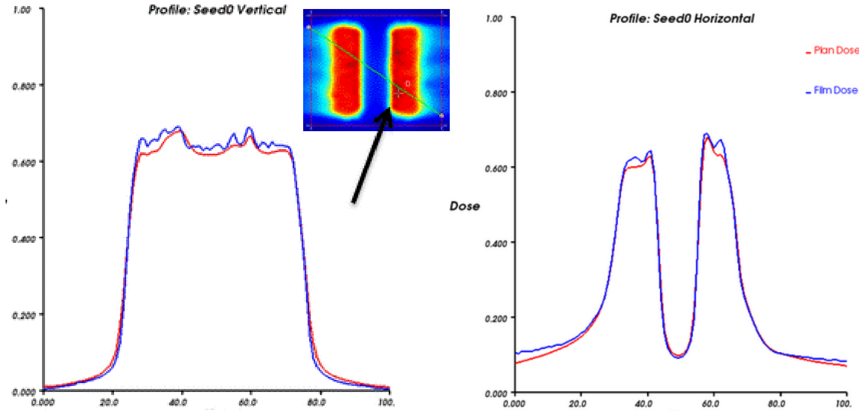

10XFFF C Shape RapidArc (low

Figure 10: Gafchromic film measurement results for the vertical and horizontal profile comparing the planned versus measured fluence in the high-dose and low-dose region for both IMRT and RapidArc plans.- The red line indicates planned dose, whereas the blue line indicates the measured dose profile. The x-axis represents the relative position of the selected profile and the y-axis presents the relative dose in percentage.

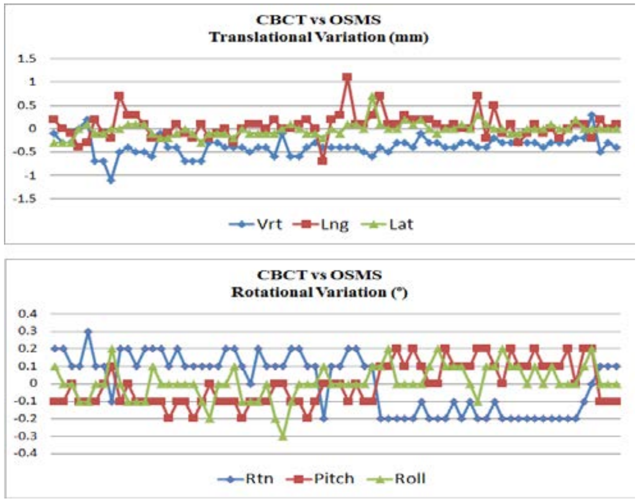
CBCT vs OSMS
Translational Variation (mm)
Vrt
Lng
Lat
CBCT vs OSMS
Rotational Variation (°)
Rtn
Pitch
Roll

(a)

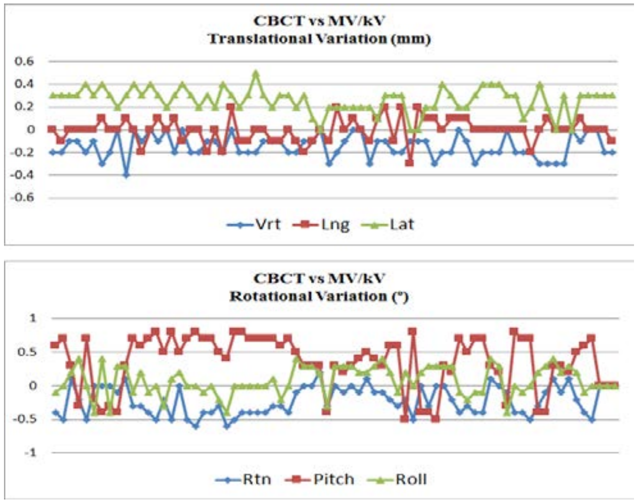
CBCT vs MV/kV
Translational Variation (mm)
Vrt
Lng
Lat
CBCT vs MV/kV
Rotational Variation (°)
Rtn
Pitch
Roll

(b)

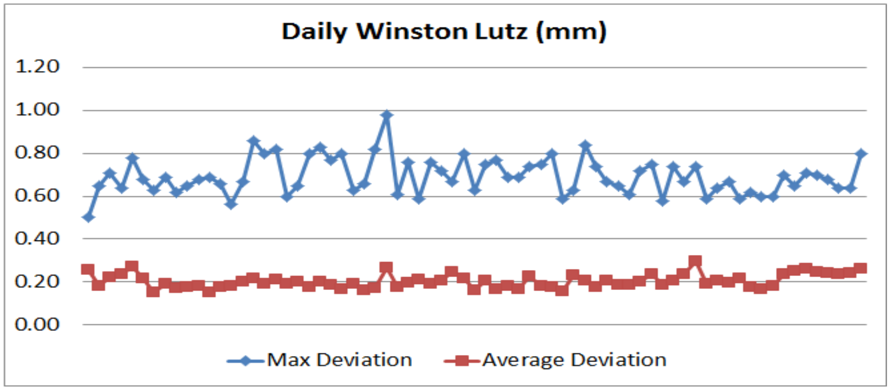
Daily Winston Lutz (mm)
1.20
1.00
0.80
0.60
0.40
0.20
0.00
Max Deviation
Average Deviation

(c)

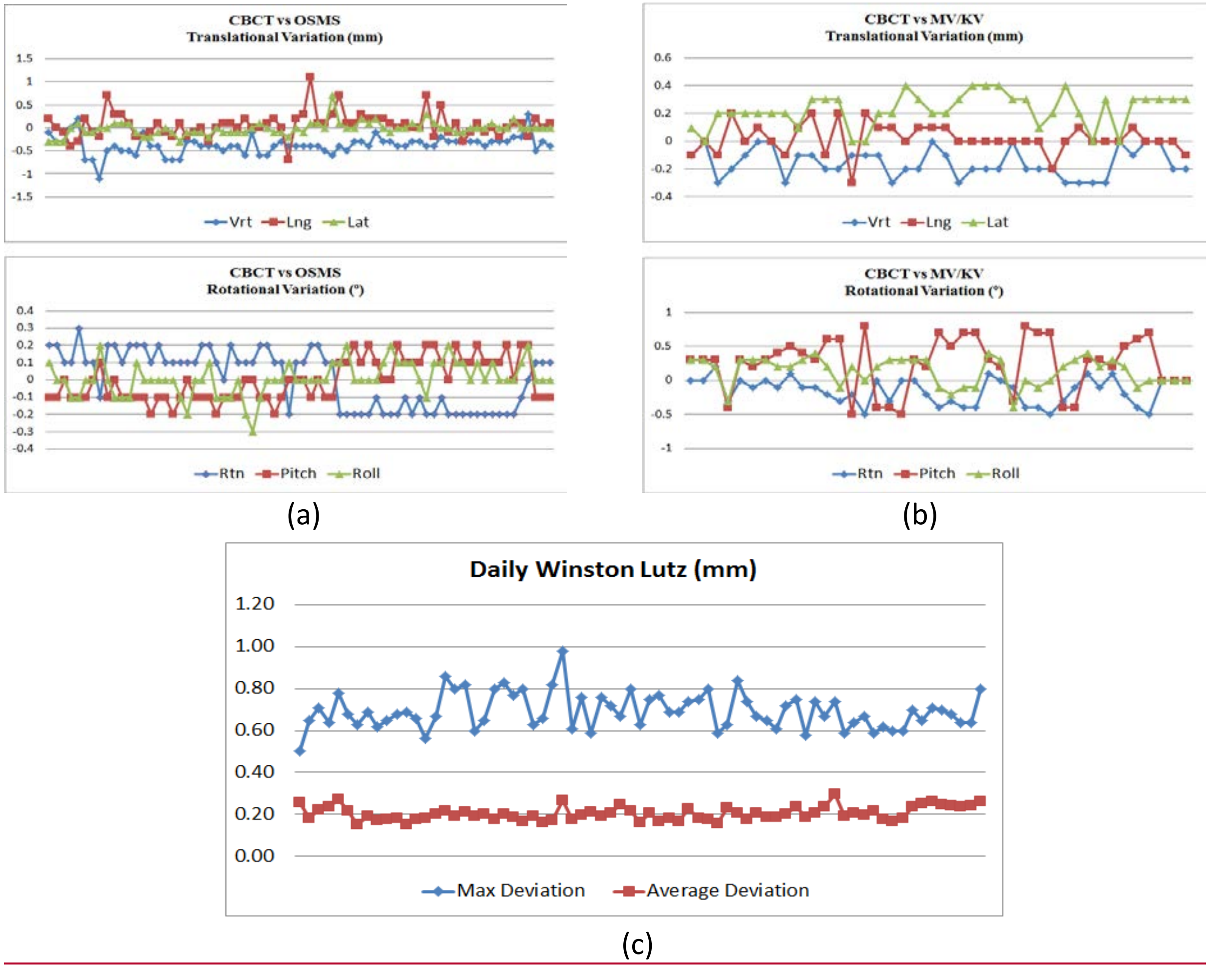

Figure 11. End-to-end testing using the OSMS QA phantom from the first three months' of operation. The daily variations of isocentric coincidence in the translational and rotational direction between the CBCT and OSMS (a) and between the CBCT and MV/k~~K~~V planar images (b). The average and maximum absolute values of the daily Winston-Lutz test performed at four gantry (0°, 90°, 180°, 270°), four couch (0°, 45°, 270°, 315°) and four collimator angles (0°, 45°, 270°, 315°) are shown in (c).

Table 1. Ion chambers and diodes used in the commissioning.

**Ion Chamber**	**Active Volume (cm^3)**	**Radius (mm)**	**Length (mm)**	**Central Electrode**	**Sensitivity (nC/Gy)**	**Tasks**
Scanditronix CC04	0.04	2.0	3.6 mm	C552	1.1	PDD, Profiles, OF ≥ 3×3 cm^2
Scanditronix CC01	0.01	1.0	3.6 mm	Steel	0.3	OF – Conical Cones
PTW PinPoint (31014)	0.015	1.0	5.0 mm	Aluminum	0.4	OF – Conical Cones
**Diode**	**Thickness of Active Volume (mm)**	**Diameter**	**Geometry – Active Area**	**Misc.**	**Sensitivity (nC/Gy)**	**Tasks**
Scanditronix SFD	0.06	0.6 mm	Circle	p-type Unshielded	5.9	PDD, Profiles, OF < 3×3 cm^2 and Conical Cones
Scanditronix PFD	0.06	2.0 mm	Circle	p-type Shielded	33.3	OF – Conical Cones
Sun Nuclear EDGE	0.0025	0.8 x 0.8 mm^2	Square	n-type Unshielded	32.0	OF – Conical Cones

Table 2. Dmax and PDD values at 5, 10, 20, and 30 cm depth for 6XFFF and 10XFFF.

Energy \ Field Size		1×1	2×2	3×3	4×4	5×5	6×6	8×8	10×10	12×12	15×15	20×20	30×30	35×35	40×40
6XFFF	**Dmax (cm)**	1.10	1.30	1.34	1.40	1.35	1.24	1.30	1.35	1.36	1.35	1.18	1.27	1.21	1.20
	**5 cm (%)**	75.8	78.5	79.9	81.2	81.8	82.4	83.3	84.2	84.4	85.2	85.3	85.8	85.9	86.2
	**10 cm (%)**	51.7	54.1	56.2	57.8	58.9	59.9	61.5	63.0	63.8	65.0	65.8	66.8	67.2	67.3
	**20 cm (%)**	25.5	26.8	28.7	29.7	30.7	31.4	32.9	34.4	35.1	36.6	37.8	39.2	39.5	39.6
	**30 cm (%)**	13.2	14.1	15.3	15.9	16.4	17.0	17.9	18.9	19.7	20.5	21.6	22.9	23.0	23.2
10XFFF	**Dmax (cm)**	1.80	2.07	2.20	2.20	2.06	2.14	2.14	2.36	2.06	1.97	2.19	1.93	1.95	2.11
	**5 cm (%)**	83.5	87.0	88.0	88.5	89.1	89.5	89.8	90.2	90.1	90.3	90.5	90.3	90.3	90.3
	**10 cm (%)**	61.2	64.3	66.3	67.2	68.2	68.7	69.5	70.6	71.0	71.4	72.0	72.2	72.5	72.5
	**20 cm (%)**	34.4	36.2	38.0	39.0	39.6	40.2	41.5	42.6	43.3	43.8	44.8	45.3	45.6	45.8
	**30 cm (%)**	20.0	21.4	22.8	23.3	23.8	24.2	25.2	25.9	26.6	26.8	27.9	28.6	28.8	29.1

Table 3. Output factors measured with CC04 and SFD for 6XFFF. The data measured by SFD was shown in bold italic type.

Y\X	1	2	3	4	5	6	7	8	10	12	15	20	25	30	35	40
**1**	***0.765***	***0.799***	***0.808***	***0.811***	***0.815***	***0.818***	***0.818***	***0.821***	***0.821***	***0.822***	***0.825***	***0.825***	***0.826***	***0.827***	***0.827***	***0.828***
**2**	***0.806***	***0.856***	***0.872***	***0.881***	***0.887***	***0.892***	***0.896***	***0.899***	***0.901***	***0.904***	***0.907***	***0.909***	***0.910***	***0.911***	***0.912***	***0.913***
**3**	***0.817***	***0.874***	0.896	0.907	0.913	0.919	0.922	0.925	0.928	0.930	0.933	0.935	0.937	0.938	0.937	0.937
**4**	***0.823***	***0.885***	0.907	0.921	0.929	0.935	0.940	0.943	0.947	0.950	0.953	0.957	0.959	0.960	0.960	0.959
**5**	***0.826***	***0.891***	0.916	0.930	0.940	0.947	0.953	0.958	0.962	0.965	0.969	0.974	0.977	0.977	0.977	0.977
**6**	***0.828***	***0.897***	0.922	0.938	0.949	0.957	0.963	0.968	0.973	0.978	0.982	0.988	0.990	0.991	0.992	0.991
**7**	***0.831***	***0.901***	0.926	0.944	0.955	0.964	0.971	0.976	0.982	0.987	0.992	0.998	1.002	1.003	1.004	1.003
**8**	***0.832***	***0.904***	0.929	0.949	0.960	0.969	0.977	0.982	0.989	0.995	1.000	1.007	1.010	1.012	1.013	1.012
**10**	***0.835***	***0.909***	0.934	0.955	0.967	0.978	0.986	0.991	1.000	1.006	1.012	1.020	1.025	1.027	1.028	1.027
**12**	***0.836***	***0.912***	0.938	0.958	0.972	0.983	0.992	0.998	1.008	1.014	1.022	1.029	1.035	1.038	1.039	1.038
**15**	***0.839***	***0.915***	0.940	0.962	0.975	0.988	0.998	1.004	1.015	1.023	1.031	1.041	1.047	1.050	1.051	1.051
**20**	***0.840***	***0.919***	0.943	0.966	0.981	0.992	1.003	1.010	1.023	1.030	1.040	1.052	1.059	1.063	1.065	1.064
**25**	***0.841***	***0.920***	0.945	0.968	0.983	0.996	1.006	1.015	1.027	1.035	1.047	1.059	1.067	1.072	1.074	1.073
**30**	***0.843***	***0.922***	0.947	0.970	0.986	0.999	1.009	1.018	1.031	1.041	1.052	1.065	1.073	1.078	1.080	1.080
**35**	***0.844***	***0.923***	0.947	0.971	0.987	1.000	1.012	1.020	1.034	1.043	1.055	1.070	1.078	1.082	1.084	1.085
**40**	***0.843***	***0.924***	0.948	0.972	0.988	1.001	1.013	1.022	1.036	1.045	1.057	1.072	1.079	1.084	1.086	1.087

Table 4. Output factors measured with CC04 and SFD for 10XFFF. The data measured by SFD was shown in bold italic type.

Y\X	1	2	3	4	5	6	7	8	10	12	15	20	25	30	35	40
**1**	***0.731***	***0.784***	***0.796***	***0.801***	***0.800***	***0.803***	***0.804***	***0.804***	***0.806***	***0.805***	***0.807***	***0.807***	***0.808***	***0.809***	***0.808***	***0.809***
**2**	***0.800***	***0.880***	***0.897***	***0.906***	***0.908***	***0.912***	***0.914***	***0.914***	***0.916***	***0.919***	***0.922***	***0.921***	***0.924***	***0.923***	***0.924***	***0.924***
**3**	***0.814***	***0.900***	0.925	0.935	0.941	0.944	0.945	0.947	0.949	0.952	0.952	0.952	0.955	0.955	0.954	0.953
**4**	***0.819***	***0.911***	0.935	0.947	0.954	0.957	0.961	0.964	0.966	0.969	0.970	0.970	0.972	0.972	0.971	0.972
**5**	***0.821***	***0.916***	0.942	0.955	0.963	0.967	0.970	0.973	0.977	0.979	0.980	0.983	0.983	0.984	0.983	0.984
**6**	***0.824***	***0.920***	0.945	0.960	0.968	0.972	0.976	0.980	0.984	0.987	0.987	0.990	0.992	0.993	0.993	0.994
**7**	***0.825***	***0.922***	0.949	0.963	0.973	0.977	0.981	0.986	0.990	0.993	0.995	0.997	0.999	1.000	1.001	1.000
**8**	***0.826***	***0.923***	0.950	0.966	0.976	0.981	0.984	0.989	0.994	0.998	1.001	1.003	1.004	1.006	1.004	1.006
**10**	***0.829***	***0.926***	0.953	0.970	0.982	0.985	0.992	0.995	1.000	1.004	1.008	1.012	1.013	1.016	1.014	1.015
**12**	***0.828***	***0.928***	0.956	0.972	0.984	0.988	0.995	0.998	1.005	1.010	1.012	1.018	1.021	1.022	1.022	1.024
**15**	***0.832***	***0.930***	0.958	0.974	0.986	0.991	0.999	1.004	1.011	1.025	1.019	1.024	1.028	1.029	1.028	1.028
**20**	***0.834***	***0.933***	0.960	0.978	0.988	0.996	1.003	1.008	1.015	1.020	1.024	1.031	1.034	1.037	1.036	1.038
**25**	***0.833***	***0.935***	0.961	0.980	0.992	0.998	1.004	1.009	1.018	1.023	1.029	1.034	1.038	1.043	1.041	1.042
**30**	***0.832***	***0.935***	0.964	0.980	0.994	0.999	1.006	1.013	1.020	1.028	1.032	1.039	1.043	1.045	1.045	1.047
**35**	***0.833***	***0.936***	0.964	0.981	0.995	1.002	1.009	1.014	1.022	1.028	1.033	1.042	1.045	1.048	1.050	1.050
**40**	***0.837***	***0.938***	0.965	0.983	0.995	1.003	1.010	1.015	1.023	1.029	1.034	1.042	1.048	1.051	1.051	1.050

Table 5. Output factors of the conical cones measured with five detectors (Edge, SFD, photon diode, CC01 and Pinpoint chamber). The measurements were shown with and without cross calibration at an intermediate field size 3 × 3 cm^2 for the Edge detector. The percent difference was calculated between OFs measured with different detectors and the data from Varian (downloaded from the Vendor website) measured with the Edge detector.

		**6X FFF**							**10X FFF**						
**Cone size (mm)**		**4**	**5**	**7.5**	**10**	**12.5**	**15**	**17.5**	**4**	**5**	**7.5**	**10**	**12.5**	**15**	**17.5**
**OF - the Edge Detector (no cross calibration)**		0.607	0.671	0.755	0.800	0.827	0.848	0.859	0.516	0.589	0.700	0.769	0.815	0.847	0.872
**OF - the Edge Detector (cross calibration at 3 × 3 cm^2)**		0.608	0.672	0.756	0.801	0.828	0.849	0.860	0.513	0.586	0.696	0.765	0.810	0.842	0.867
**% diff of measured vs. Varian representative OF**	**Edge**	0.8	1.2	0.1	0.8	0.5	0.5	1.0	0.2	0.5	-0.7	0.1	0.0	-0.4	1.3
	**SFD**	0.3	-1.3	-3.0	-2.2	-1.9	-1.5	-0.5	1.0	-0.9	-3.7	-3.2	-2.8	-2.7	-0.7
	**Photon diode**	-7.1	-2.6	-1.0	0.2	0.2	0.1	0.9	-8.1	-3.7	-2.2	-1.3	-0.9	-1.1	0.6
	**CC01**	-36.0	-24.3	-9.5	-4.8	-2.9	-1.7	-0.1	-34.1	-23.9	-11.6	-7.2	-4.9	-3.7	-1.0
	**Pinpoint**	-43.6	-32.1	-14.3	-7.0	-4.0	-2.6	-0.7	-42.7	-32.3	-17.1	-10.2	-7.0	-5.4	-2.6

Table 6. The distance between the BB center and the isocenter after couch pitch and roll positioning. I: Inferior; S: Superior; L: Lleft; R: Right. I 0.2 means the BB was 0.2 mm inferiorly from the isocenter.

	Distance [mm]							
	No Weight				***With Weight (96.2 kg)***			
Pitch/Roll	***MV AP***		***KV RT Lat***		***MV AP***		***KV RT Lat***	
0°/0°	0.0	0.0	0.0	L 0.3	S 0.1	0.0	S 0.1	0.0
+3°/+3°	I 0.2	L 0.2	0.0	L 0.4	0.0	L 0.4	I 0.2	0.0
-3°/-3°	S 0.1	R 0.1	0.0	0.0	S 0.3	R 0.3	S 0.5	0.0
0°/0°	0.0	0.0	0.0	L 0.2	S 0.1	0.0	S 0.1	L 0.2

Table 7. Composite Gafchromic film and ion chamber results for the measurements in the high-dose and low-dose region for both IMRT and RapidArc plans.

	6XFFF		10XFFF	
**Plan**	**Global Gamma 3%/3mm**	**Point Dose (Percent Difference)**	**Global Gamma 3%/3mm**	**Point Dose (Percent Difference)**
Hard C IMRT (PTV)	90.6	3.5%	90.0	4.7%
Hard C IMRT (low dose)	87.4	2.4%	91.1	11.9%
Hard C RA (PTV)	93.0	-0.5%	97.5	-0.1%
Hard C RA (low dose)	95.2	-4.2%	98.7	2.4%
HN IMRT (PTV)	94.1	1.0%	98.1	2.5%
HN IMRT (low dose)	97.0	0.4%	99.5	1.3%
HN RA (PTV)	97.9	0.4%	98.7	-0.4%
HN RA (low dose)	98.1	0.2%	98.4	-0.2%
HN SIB IMRT (PTV)	97.5	-0.9%	98.9	1.0%
HN SIB IMRT (low dose)	98.6	-0.2%	97.5	1.7%
HN SIB RA (PTV)	99.0	-0.4%	98.1	-0.7%
HN SIB RA (low dose)	98.1	0.7%	97.8	0.5%
Prostate IMRT (PTV)	95.7	-1.8%	98.2	-0.1%
Prostate IMRT (low dose)	89.6	-3.1%	95.3	-0.2%
Prostate RA (PTV)	99.1	-1.0%	99.3	0.0%
Prostate RA (low dose)	95.7	-3.1%	99.4	-1.9%
Prostate LN IMRT (PTV)	86.2	-0.9%	99.0	-0.5%
Prostate LN IMRT (low dose)	96.2	-0.8%	98.9	-0.2%
Prostate LN RA (PTV)	98.7	0.6%	99.2	0.4%
Prostate LN RA (low dose)	99.0	1.7%	97.9	1.6%
SIMT RA (low dose)	98.9	2.9%	100.0	-0.7%

Table 8. Summary of IROC phantom irradiation results for the lung and spine phantoms.

Phantom	***TLD Location***	***IROC vs Inst***	***Criteria***	***Film Plane***	***Gamma Index***	***Criteria***
Lung Phantom	PTV_TLD_sup	0.97	0.92-1.02	Axial	100%	≥80%
				Coronal	100%	≥80%
	PTV_TLD_inf	0.98	0.92-1.02	Sagittal	100%	≥80%
Spine Phantom	PTV_TLD_sup_ant	1.01	0.93-1.07	Axial	90%	≥85%
	PTV_TLD_inf_ant	1.00	0.93-1.07			
	PTV_TLD_sup_post	1.00	0.93-1.07	Sagittal	91%	≥85%
	PTV_TLD_inf_post	0.99	0.93-1.07			
